# Pharmacological therapies for neglected tropical diseases: a systematic review and evidence gap mapping

**DOI:** 10.1590/0037-8682-0056-2026

**Published:** 2026-07-03

**Authors:** Julia Barbosa Domingues da Silva, Fernando Fernandez-Llimos, Helena Hiemisch Lobo Borba

**Affiliations:** 1 Universidade Federal do Paraná, Programa de Pós-Graduação em Ciências Farmacêuticas, Curitiba, PR, Brasil.; 2 Universidade do Porto, Faculdade de Farmácia, Porto, Portugal.

**Keywords:** Global health, Neglected diseases, Drug therapy, Systematic review, Evidence gaps

## Abstract

**Background:**

Neglected tropical diseases (NTDs) comprise 21 endemic infections that affect over one billion people worldwide. This study mapped and identified evidence gaps regarding pharmacological therapies for all WHO-recognized NTDs.

**Methods:**

We systematically searched PubMed for systematic reviews (SRs) and randomized controlled trials (RCTs), and the ClinicalTrials.gov database for ongoing RCTs. Methodological quality was assessed using the AMSTAR-2 for SRs and the Cochrane Risk of Bias tool for RCTs. Data were synthesized into evidence and gap maps, and the distribution of evidence was analyzed according to quality.

**Results:**

Twenty-four maps were developed, including 74 SRs, 500 RCTs, and 70 ongoing clinical trials; of these, only seven SRs and 17 RCTs were rated as high quality or as having a low risk of bias, respectively. Trachoma exhibited the highest number of evidence gaps, while Buruli ulcer had the lowest. Taeniasis/cysticercosis presented the highest proportion (31.8%) of high-quality SRs, whereas Chagas disease showed the highest proportion of RCTs with a low risk of bias (18.4%).

**Conclusions:**

Significant gaps remain in the evidence regarding the efficacy and safety of pharmacological treatments for WHO-recognized NTDs. The currently available evidence is predominantly of low methodological quality and is often insufficient to support robust therapeutic conclusions, highlighting the urgent need for rigorously designed clinical trials.

## INTRODUCTION

The World Health Organization (WHO) defines neglected tropical diseases (NTDs) as a diverse group of 21 infectious conditions that disproportionately affect impoverished populations in tropical and subtropical regions of Africa, Asia, and Latin America. These diseases are caused by a wide range of pathogens, including viruses, bacteria, parasites, fungi, and toxins, and constitute a major public health challenge^1^. Many NTDs are vector-borne, involve animal reservoirs, and have complex life cycles, which further complicates control and eradication efforts^2^.

The global burden of NTDs is substantial. The WHO estimates that over 1.4 billion people require preventive or therapeutic interventions for at least one NTD annually. Beyond their immediate clinical consequences, NTDs perpetuate cycles of poverty and stigma. They are associated with considerable mortality and morbidity, causing approximately 120,000 deaths and 14.1 million disability-adjusted life years annually^1^. Chronic sequelae, including long-term disability and malnutrition, markedly impair work capacity and child development, resulting in substantial economic consequences for endemic countries^3^.

Despite their complexity, many NTDs are preventable through established public health measures. Safe water, sanitation, and hygiene interventions are critical for controlling diseases, such as trachoma and schistosomiasis^4^, whereas vector control remains essential for dengue, Chagas disease, and lymphatic filariasis^1^. However, prevention alone is insufficient. Effective management also requires equitable access to reliable diagnostics and pharmacological treatments with demonstrated safety and efficacy.

A major challenge is the limited investment in therapeutic innovation for NTDs. The pharmaceutical industry has historically allocated few resources to research and development for these diseases, largely because of their perceived low commercial returns. Consequently, evidence regarding pharmacological therapies remains fragmented, and available treatments may be outdated, toxic, or insufficiently supported by high-quality data^5^. In this context, evidence and gap maps (EGMs) are valuable tools for systematically visualizing the distribution of existing evidence, identifying areas of research saturation, and highlighting priority areas for future funding and innovation^6^.

Thus, this study aimed to map and identify gaps in the available evidence regarding pharmacological therapies for all 21 WHO-recognized NTDs.

## METHODS

### Study design

A systematic review was conducted following the JBI Manual for Evidence Synthesis guidelines^7^, and the EGMs were constructed in accordance with the Campbell Collaboration recommendations^6^. This study protocol was registered with PROSPERO (CRD42023440846).

### Literature selection

To identify eligible SRs and RCTs, we searched PubMed, which includes MEDLINE, PubMed Central, and indexed articles from the Cochrane Database of Systematic Reviews, JBI Evidence Synthesis, and Campbell Systematic Reviews. Searches were updated through October 6, 2024. Given the exceptionally broad scope of this study, which encompassed 21 neglected tropical diseases and generated a large volume of records, a pragmatic decision was made to focus the bibliographic search on PubMed and ClinicalTrials.gov to ensure operational feasibility. This approach leveraged PubMed’s extensive coverage of global health and infectious disease literature. To mitigate the limitations associated with restricting the number of databases, we employed a highly sensitive and comprehensive search strategy combining specific MeSH terms and free-text keywords for each disease, followed by rigorous screening procedures. The complete search strategies are presented in .

Eligible studies included SRs of clinical trials or observational studies and RCTs that assessed the efficacy or safety of pharmacological treatments containing active substances for the etiological or symptomatic management of the 21 WHO-recognized NTDs. Interventional Phases I-III (pre-marketing) trials were included, whereas Phase IV studies were excluded to maintain methodological consistency and comparability. Studies evaluating medicinal plants, complementary therapies, or non-pharmacological procedures or devices were excluded. No restrictions were applied regarding language or publication date.

To provide a forward-looking perspective, ongoing RCTs were retrieved from ClinicalTrials.gov using the specific name of each NTD in the “Condition or disease” search field. Trials with a status of “completed,” “terminated,” “suspended,” “withdrawn,” or “unknown” were excluded from the analysis of ongoing trials.

Retrieved records were first screened by titles and abstracts. Articles considered potentially eligible during the initial screening were reviewed in full to determine eligibility, and data were extracted from studies that met the inclusion criteria. Screening was conducted by the primary researcher and was systematically verified by a second reviewer, who evaluated 100% of the included and excluded records. Disagreements between reviewers were resolved through consensus meetings.

### Data extraction and synthesis

Data extraction was initially performed by one researcher using standardized Excel forms. Subsequently, all extracted data were systematically cross-checked against the original full-text articles by a second independent reviewer to ensure accuracy and completeness. Discrepancies identified during data extraction were resolved by consensus.

Extracted variables included study metadata, such as authors, year, location, and funding; population characteristics, including sample size, age, sex, and disease severity; intervention details, including drug, dosage, and regimen; and outcomes, including efficacy, safety, and adverse events. To facilitate visualization within the EGMs, related specific outcomes were grouped into broader overarching categories. A comprehensive data dictionary specifying which individual outcomes were aggregated into each broad category is provided in .

The methodological quality of SRs was evaluated using AMSTAR-2^8^. RCTs were assessed for risk of bias using the original Cochrane Risk of Bias tool (RoB 1)^9^. This version was selected because the analysis was conducted at the study level, with multiple outcomes grouped into broader categories to fit the EGM framework. Because RoB 2 requires outcome-specific assessments linked to individual effect estimates, its application was not feasible within this aggregated structure.

Data were synthesized to generate EGMs for each of the 21 NTDs, mapping available treatments against the main outcomes of interest. For clarity, related outcomes, such as cardiovascular parameters, were grouped into broader categories.

The evidence landscape was analyzed by calculating the distribution of studies across interventions and outcomes, and by determining the proportion of evidence across all methodological quality and risk of bias categories relative to the number of outcomes multiplied by the number of interventions in each EGM.

## RESULTS

**FIGURE 1:**
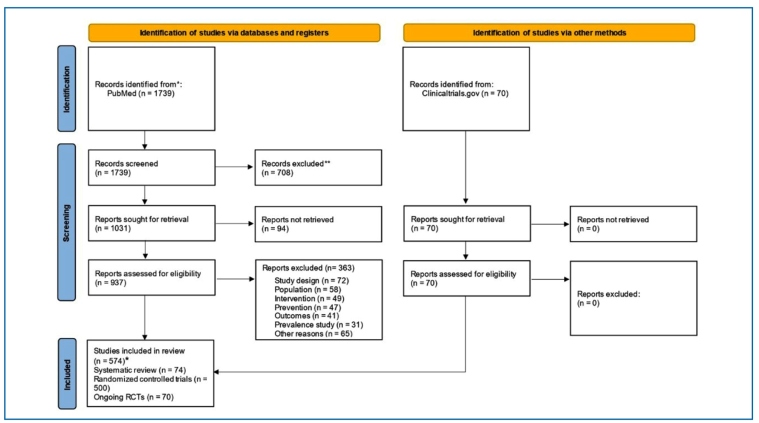
PRISMA flow diagram^14^. **Note:** *The number of studies included for each disease is shown in Table 1.

An aggregated PRISMA flow diagram summarizing the overall literature search and study selection process is presented in Figure 1. Although this diagram represents the total pool of literature evaluated across all conditions, the searches and study selection procedures were conducted separately for each NTD. Table 1 and detail the volume of retrieved literature, including the number of studies excluded during the screening and eligibility phases. Furthermore, both the detailed data extraction tables and the comprehensive list of studies excluded after full-text review, with reasons for exclusion, are available in the Open Science Framework (OSF) repository (https://doi.org/10.17605/OSF.IO/MC39U).

**TABLE 1:** Number of included studies by study design.

Neglected tropical disease	N included SRs	N included RCTs	N included RCT-O
Buruli ulcer	3	5	2
Chagas disease	6	13	7
Dengue and chikungunya	2	30	20
Dracunculiasis (Guinea-worm disease)	0	2	0
Echinococcosis	4	7	0
Foodborne trematodiases	0	5	1
Human African trypanosomiasis (sleeping sickness)	3	14	2
Leishmaniasis	13	130	11
Leprosy (Hansen’s disease)	4	43	4
Lymphatic filariasis	7	52	1
Mycetoma, chromoblastomycosis, and other deep mycoses	1	1	0
Onchocerciasis (river blindness)	1	21	4
Podoconiosis	0	0	0
Rabies	1	14	6
Scabies and other ectoparasitoses	4	27	5
Schistosomiasis	6	43	1
Snakebite envenoming	2	12	1
Soil-transmitted helminthiases	5	37	3
Taeniasis/cysticercosis	7	25	1
Trachoma	5	16	0
Yaws and other endemic treponematoses	0	3	1

**Note: N:** number; **RCT:** randomized controlled trial; **RCT-O:** ongoing randomized controlled trial; **SR:** systematic review.

A total of 24 EGMs were generated, covering the 21 WHO-recognized NTDs. The final synthesis comprised 74 SRs, 500 RCTs, and 70 ongoing clinical trials (). To enhance accessibility and facilitate navigation of disease-specific data points, complete high-resolution versions of all 24 EGMs are also available in the OSF repository (https://doi.org/10.17605/OSF.IO/MC39U).

The methodological quality assessment revealed substantial limitations. Among the SRs, 43 were rated as critically low, 18 as low, six as moderate, and only seven as high quality. Similarly, the risk of bias assessment of the RCTs showed that 323 trials had a high risk of bias, 160 had an unclear risk of bias, and only 17 trials (3.4%) had a low risk of bias.

Overall, 511 interventions and 193 outcomes were mapped. The evidence base varied markedly across conditions: the median number of interventions per disease was 19 (range: 2-70), and the median number of outcomes was 7 (range: 3-16). Cutaneous leishmaniasis had the highest volume of retrieved studies, whereas dracunculiasis had the lowest. No eligible studies were identified for podoconiosis.

Table 2 presents the distribution of interventions, outcomes, and the proportion of high-quality evidence for each disease. This analysis revealed extensive gaps in efficacy and safety data, with most available evidence classified as moderate to low quality. When quality density was examined, defined as the proportion of high-quality studies among the total number of studies for each disease, taeniasis/cysticercosis had the highest density of high-quality SRs (31.8%), followed by scabies (20.8%) and lymphatic filariasis (9.6%). For RCTs, Chagas disease, specifically etiological treatment, had the highest density of trials with a low risk of bias (18.4%), followed by soil-transmitted helminthiases (8.2%) and dengue/chikungunya symptomatic treatment (6.8%).

**TABLE 2:** Interventions, outcomes, and proportion of high-quality evidence by disease.

Neglected tropical disease	N interventions	N outcomes	% High quality	
			SR (%)	RCT (%)
**Etiological treatment**				
Buruli ulcer	19	6	0	0
Chagas disease	7	7	0	50
Dengue and chikungunya	13	7	NA	0
Dracunculiasis (Guinea-worm disease)	2	3	NA	0
Echinococcosis	7	10	0	0
Foodborne trematodiases	7	5	NA	0
Human African trypanosomiasis (sleeping sickness)	11	7	0	0
Leishmaniasis (cutaneous)	70	11	0	2.1
Leishmaniasis (visceral)	27	11	0	0
Leprosy (Hansen’s disease)	59	16	0	7
Lymphatic filariasis	20	13	14.3	0
Mycetoma, chromoblastomycosis, and other deep mycoses	27	5	0	0
Onchocerciasis (river blindness)	22	9	0	0
Podoconiosis	-	-	NA	NA
Rabies	20	5	0	0
Scabies and other ectoparasitoses	16	12	50	0
Schistosomiasis	28	10	16.7	0
Snakebite envenoming	4	5	0	0
Soil-transmitted helminthiases	40	7	0	13.5
Taeniasis/cysticercosis	18	11	42.9	0
Trachoma	49	9	0	6.3
Yaws and other endemic treponematoses	3	6	NA	0				
**Symptomatic treatment**				
Chagas disease	11	6	NA	14.3
Dengue and chikungunya	19	7	0	18.2
Snakebite envenoming	10	5	0	0

**Note: N:** number; **NA:** not applicable; **RCT:** randomized controlled trial; **SR:** systematic review. RCTs with a low risk of bias were considered to be of high quality.

Regarding future research activity, yaws had the highest density of ongoing RCTs relative to completed trials (27.8%), followed by snakebite envenoming (25%) and dengue/chikungunya vaccines (24%). Table 3 provides further details on the density of identified gaps across intervention-outcome pairs.

**TABLE 3:** Density of evidence across interventions and outcomes.

Neglected tropical disease	Out	Int	IxO	SR				RCT			Ong (%)
				H (%)	M (%)	L (%)	CL (%)	H Rob (%)	M Rob (%)	L Rob (%)	
Buruli ulcer	6	19	114	0	70.2	0	49.1	13.2	3.5	0	8.8
Chagas disease (antitrypanosomal)	7	7	49	0	20.4	42.9	8	12.2	0	18.4	14
Chagas disease (symptomatic)	6	11	66	NA	NA	NA	NA	16.7	9.1	6.1	7.6
Dengue and chikungunya (symptomatic)	7	19	133	0	0	0	5.3	8.3	8.3	6.8	13.5
Dengue and chikungunya (vaccines)	7	13	91	NA	NA	NA	NA	9.9	8.8	0	24
Dracunculiasis (Guinea-worm disease)	3	2	6	NA	NA	NA	NA	16.7	50	0	NA
Echinococcosis	10	7	70	0	0	17	36	24.3	0	0	NA
Foodborne trematodiases	5	7	35	NA	NA	NA	NA	57.1	0	0	8.6
Human African trypanosomiasis (sleeping sickness)	7	11	77	0	45.5	10.4	0	49.4	10.4	0	3.9
Leishmaniasis (cutaneous)	11	70	770	0	0	19.1	5.8	16.4	9.7	0.9	3.5
Leishmaniasis (visceral)	11	27	297	0	0	0	17.8	22.2	1.3	0	3.4
Leprosy (Hansen’s disease)	16	59	944	0	0	3.2	9.5	10.2	4.7	0.8	2.6
Lymphatic filariasis	13	20	260	9.6	0	9.6	8.1	15	25	0	6.2
Mycetoma, chromoblastomycosis, and other deep mycoses	5	27	135	0	55.6	0	0	0	5.9	0	NA
Onchocerciasis (river blindness)	9	22	198	0	3	0	0	15.7	25.8	0	13.1
Podoconiosis	-	-	-	NA	NA	NA	NA	NA	NA	NA	NA
Rabies	5	18	90	0	0	0	2.2	25.6	11.1	0	23.3
Scabies and other ectoparasitoses	12	16	192	20.8	0	4.2	1.6	21.4	12	0	3.6
Schistosomiasis	10	28	280	4.3	0	4.3	11.4	23.2	16.4	0	1.8
Snakebite envenoming	5	4	20	0	0	0	5	25	10	0	25
Snakebite envenoming (anti-venom serum)	5	10	50	0	0	0	8	8	14	0	NA
Soil-transmitted helminthiases	7	40	280	0	0	5.4	25.7	20	20.7	8.2	2.9
Taeniasis/cysticercosis	11	18	198	31.8	0	7.6	14.6	21.7	14.1	0	4
Trachoma	9	49	441	0	0	7.5	11.6	4.5	2	0.9	NA
Yaws and other endemic treponematoses	6	3	18	NA	NA	NA	NA	27.8	0	0	27.8

**Note: NA**: not applicable; **Out** outcomes; **Int:** interventions; **IxO** number of outcomes multiplied by the number of interventions; **SR:** systematic review; **RCT:** randomized controlled trial; **Ong:** ongoing clinical trial; For SR: **H:** high confidence; **M:** moderate confidence; **L:** low confidence; **CL:** critically low confidence; For RCT: **H RoB:** high risk of bias; **M RoB:** moderate risk of bias; **L RoB:** low risk of bias. Percentages for quality assessments and risk of bias were calculated using the number of outcomes (Out) multiplied by the number of interventions (Int) in each map (IxO) as the denominator.

## DISCUSSION

This study provides a comprehensive map of the pharmacological evidence landscape across all 21 WHO-recognized NTDs. The findings reveal a critical disconnect between the volume and the quality of available evidence. Although some diseases have accumulated a substantial body of literature, this evidence is often fragmented, methodologically limited, or redundant. These findings indicate that a high number of publications does not necessarily translate into robust therapeutic guidance, generating an “illusion of abundance” for conditions such as leishmaniasis.

Despite the value of EGMs for guiding policy, identifying research priorities, and informing funding decisions^6^, their application in NTD research remains limited. Our analysis highlights a concerning misalignment between research output and disease burden. For instance, while leishmaniasis accounted for the highest number of studies, nearly half of all intervention-outcome combinations remained unaddressed. Conversely, high-burden diseases, such as Chagas disease and trachoma lack sufficient high-quality evidence to support treatment guidance. This imbalance suggests a pattern of research waste, whereby research efforts are repeatedly directed toward already studied areas rather than toward unresolved and clinically important knowledge gaps.

The limited number of active clinical trials reflects the persistent market failure in NTD research and development. Although the 2012 London Declaration and subsequent medicine donation programs expanded access to treatments in endemic regions^10^, investment in NTD research remains disproportionately low, accounting for only 1.9% of global research and development funding in 2022^11^. This global pattern is also evident at the national level. In Brazil, Melo et al. (2023) demonstrated a similar disconnect between disease prevalence and funding allocation, with high-prevalence conditions, such as Chagas disease and chikungunya receiving limited funding compared with dengue^12^. Our findings further indicate that, where trials exist, they predominantly evaluate repurposed established drugs rather than developing novel chemical entities. This lack of therapeutic innovation may hinder progress toward the targets outlined in the WHO Roadmap^2^.

The pervasive low quality of available evidence is particularly concerning. Most SRs were rated as critically low quality using AMSTAR-2, often because of limitations in fundamental domains, such as comprehensive search strategies and risk of bias assessments^8^. Similarly, fewer than 4% of RCTs had a low risk of bias, with common methodological shortcomings related to blinding and risks of performance and detection bias^6^. These findings are consistent with Altman’s warning regarding poor medical research, suggesting that limited resources may be spent on studies that are insufficiently designed to inform clinical practice reliably^13^.

This study has several limitations. First, the search strategy was restricted to PubMed and ClinicalTrials.gov. This pragmatic decision was made because of the exceptionally broad scope of the study and the large volume of available literature. Although PubMed provides extensive coverage of high-impact global health literature and has substantial indexing overlap with other databases, excluding other major platforms (e.g., EMBASE, CENTRAL) and regional databases (e.g., LILACS, African Index Medicus), may have led to the omission of locally published trials, particularly from highly endemic regions, such as Latin America and Africa. Second, we used RoB 1 to assess risk of bias at the study level. Although this approach was appropriate for the broad scope of the mapping, it provides less granularity than outcome-specific assessments (e.g., RoB 2). In addition, outcomes were grouped into broader categories to improve visual clarity in the evidence maps, which may have obscured specific nuances in efficacy and safety data. Consequently, study-level risk of bias assessment may have limited the ability to detect outcome-specific sources of bias, particularly those related to measurement and selective reporting.

Overall, these findings underscore the need to shift the current research paradigm from generating “more research” to producing “better research.” The current pharmacological evidence base for NTDs remains insufficient to support robust clinical guidance for many conditions. Future investment should prioritize rigorously designed, high-quality clinical trials and novel drug discovery, rather than increasing the number of low-quality trials or redundant SRs.

## Data Availability

Research data is available in the Supplementary material and in the Open Science Framework repository (https://doi.org/10.17605/OSF.IO/MC39U).

## References

[1] World Health Organization (WHO) (2020). Neglected tropical disease.

[2] World Health Organization (WHO) (2020). Ending the neglect to attain the Sustainable Development Goals: a road map for neglected tropical diseases 2021-2030.

[3] Mukherjee S (2023). The United States Food and Drug Administration (FDA) regulatory response to combat neglected tropical diseases (NTDs): A review. PLoS Negl Trop Dis.

[4] Velleman Y, Wicken J (2015). WASH: The silent weapon against NTDs Working together to achieve prevention, control and elimination.

[5] Wise J (2019). Spending on research into neglected disease reached record high in 2017. BMJ.

[6] White H, Albers B, Gaarder M, Kornør H, Littell J, Marshall Z (2020). Guidance for producing a Campbell evidence and gap map. Campbell Syst Rev.

[7] Aromataris E, Lockwood C, Porritt K, Pilla B, Jordan Z (2024). JBI Manual for Evidence Synthesis: JBI Evid Synth.

[8] Li L, Asemota I, Liu B, Gomez-Valencia J, Lin L, Arif AW (2022). AMSTAR 2 appraisal of systematic reviews and meta-analyses in the field of heart failure from high-impact journals. Syst Rev.

[9] Higgins JP, Altman DG, Gøtzsche PC, Jüni P, Moher D, Oxman AD (2011). The Cochrane Collaboration's tool for assessing risk of bias in randomised trials. BMJ.

[10] Bradley M, Taylor R, Jacobson J, Guex M, Hopkins A, Jensen J (2021). Medicine donation programmes supporting the global drive to end the burden of neglected tropical diseases. Trans R Soc Trop Med Hyg.

[11] G-Finder (2024). Neglected disease research and development: The higher cost of lower funding.

[12] Melo GBT, Angulo-Tuesta A, Silva END, Santos TDS, Uchimura LYT, Obara MT (2023). Evolution of research funding for neglected tropical diseases in Brazil, 2004-2020. PLoS Negl Trop Dis.

[13] Altman DG (2002). Poor-quality medical research: what can journals do?. JAMA.

[B14] Page MJ, McKenzie JE, Bossuyt PM, Boutron I, Hoffmann TC, Mulrow CD (2021). The PRISMA 2020 statement: an updated guideline for reporting systematic reviews. BMJ.

