# Supplementary material for: Pharmacological therapies for neglected tropical diseases: a systematic review and evidence gap mapping
Source: Rev Soc Bras Med Trop. 2026 Jul 3;59:e0056-2026. doi: 10.1590/0037-8682-0056-2026 (PMC13331190; doi:10.1590/0037-8682-0056-2026)
Supplement: Supplementary Table 1 [file 1678-9849-rsbmt-59-e0056-2026-md1.pdf]

Supplementary Table 1. Search strategies for PubMed

#1	"Drug Therapy"[MeSH Terms] OR ("drug"[Title/Abstract] AND "therapy"[Title/Abstract]) OR "Pharmacotherapy"[Title/Abstract] OR "Pharmaceutical Preparations"[MeSH Terms] OR ("Pharmaceutical"[Title/Abstract] AND ("Product"[Title/Abstract] OR "Preparation"[Title/Abstract])) OR "Medication"[Title/Abstract]
For SR	
#2	"meta analysis"[Publication Type] OR "Meta-Analysis as Topic"[MeSH Terms] OR "Systematic Reviews as Topic"[MeSH Terms] OR "systematic review"[Title/Abstract] OR "meta analysis"[Title/Abstract] OR "metanalysis"[Title/Abstract] OR "systematic review"[Publication Type]
For RCTs	
#2	"Randomized Controlled Trial"[Publication Type] OR "Randomized Controlled Trials as Topic"[MeSH Terms] OR ("clinical"[Title/Abstract] AND ("trial"[Title/Abstract] OR "study"[Title/Abstract]) AND ("random*"[Title/Abstract] OR "random allocation"[MeSH Terms]))
For Buruli ulcer	
#3	"Buruli Ulcer"[MeSH Terms] OR (("Buruli"[Title/Abstract] OR "Bairnsdale"[Title/Abstract] OR "Searls"[Title/Abstract] OR "Daintree"[Title/Abstract] OR "Mossman"[Title/Abstract]) AND ("Ulcer"[All Fields] OR "disease"[Title/Abstract])) OR "Mycobacterium ulcerans"[MeSH Terms] OR "Mycobacterium ulcerans"[Title/Abstract]
For Chagas disease	
#3	"Chagas Disease"[MeSH Terms] OR ("Chagas"[Title/Abstract] AND ("Disease"[Title/Abstract] OR "infection"[Title/Abstract])) OR "American Trypanosomiasis"[Title/Abstract] OR "Trypanosoma cruzi"[MeSH Terms] OR

	"Trypanosoma cruzi"[Title/Abstract] OR "kissing bug"[Title/Abstract] OR "Triatominae"[MeSH Terms] OR "Triatominae"[Title/Abstract] OR "Panstrongylus"[Title/Abstract] OR "Rhodnius"[Title/Abstract] OR "Triatoma"[Title/Abstract]
For Dengue and chikungunya	
#3	"Dengue"[MeSH Terms] OR "Dengue"[Title/Abstract] OR (("Breakbone"[Title/Abstract] OR "break bone"[Title/Abstract] OR "break bone"[Title/Abstract]) AND ("Fever"[Title/Abstract] OR "disease"[Title/Abstract])) OR "chikungunya"[Title/Abstract] OR "Chikungunya Fever"[MeSH Terms] OR "Chikungunya virus"[MeSH Terms]
For Dracunculiasis (Guinea-worm disease)	
#3	"Dracunculiasis"[MeSH Terms] OR "Dracunculiasis"[Title/Abstract] OR "guinea worm"[Title/Abstract] OR "guinea worm"[Title/Abstract] OR "Dracunculosis"[Title/Abstract] OR "Dracunculus Nematode"[MeSH Terms] OR "Dracunculus medinensis"[Title/Abstract]
For Echinococcosis	
#3	"Echinococcosis"[MeSH Terms] OR "Echinococcosis"[Title/Abstract] OR "Echinococcus"[Title/Abstract] OR "Hydatidosis"[Title/Abstract] OR "Hydatid"[Title/Abstract]
For Foodborne trematodiases	
#3	("foodborne"[Title/Abstract] AND ("trematodiases"[Title/Abstract] OR "trematode"[Title/Abstract])) OR (("liver"[Title/Abstract] OR "parasitic"[Title/Abstract]) AND "flukes"[Title/Abstract]) OR "Clonorchis"[Title/Abstract] OR "Opisthorchis"[Title/Abstract] OR "Fasciola"[Title/Abstract] OR

	"Paragonimus"[Title/Abstract] OR "Clonorchiasis"[Title/Abstract] OR "Opisthorchiasis"[Title/Abstract] OR "Fasciolasis"[Title/Abstract] OR "Paragonimiasis"[Title/Abstract]
For Human African trypanosomiasis (sleeping sickness)	
#3	"trypanosomiasis, african"[MeSH Terms] OR ("Trypanosoma"[Title/Abstract] AND ("gambiense"[Title/Abstract] OR "brucei"[Title/Abstract] OR "rhodesiense"[Title/Abstract])) OR "African Trypanosomiasis"[Title/Abstract] OR "Sleeping Sickness"[Title/Abstract] OR "Nagana"[Title/Abstract]
For Leishmaniasis	
#3	"Leishmaniasis"[MeSH Terms] OR "Leishmaniasis"[Title/Abstract] OR "Leishmania"[Title/Abstract]
For Leprosy (Hansen's disease)	
#3	"Leprosy"[MeSH Terms] OR "Leprosy"[Title/Abstract] OR (("Hansen's"[Title/Abstract] OR "Hansen"[Title/Abstract]) AND "Disease"[Title/Abstract]) OR "Mycobacterium leprae"[MeSH Terms] OR "Mycobacterium leprae"[Title/Abstract] OR "Mycobacterium lepromatosis"[Supplementary Concept] OR "Mycobacterium lepromatosis"[Title/Abstract]
For Lymphatic filariasis	
#3	"elephantiasis, filarial"[MeSH Terms] OR (("Filarial"[Title/Abstract] OR "Filariasis"[Title/Abstract] OR "Filaria"[Title/Abstract]) AND ("Lymphatic"[Title/Abstract] OR "Bancroftian"[Title/Abstract] OR "bancrofti"[Title/Abstract] OR "Malayi"[Title/Abstract] OR "Elephantiasis"[Title/Abstract])) OR "Wuchereria bancrofti"[MeSH Terms] OR "Wuchereria bancrofti"[Title/Abstract] OR "Brugia

	malayi"[MeSH Terms] OR ("Brugia"[Title/Abstract] AND ("Malayi"[Title/Abstract] OR "malayus"[Title/Abstract] OR "timori"[Title/Abstract]))
For Mycetoma, chromoblastomycosis and other deep mycoses	
#3	"Mycetoma"[MeSH Terms] OR "Mycetoma"[Title/Abstract] OR "Maduromycosis"[Title/Abstract] OR "Madura Foot"[Title/Abstract] OR "Actinomycetoma"[Title/Abstract] OR "Eumycetoma"[Title/Abstract] OR "Chromoblastomycosis"[MeSH Terms] OR "Chromoblastomycosis"[Title/Abstract] OR "Dermatitis Verrucosa"[Title/Abstract] OR "Chromomycosis"[Title/Abstract]
For Onchocerciasis (river blindness)	
#3	"onchocerciasis, ocular"[MeSH Terms] OR "River Blindness"[Title/Abstract] OR "Ocular Onchocerciasis"[Title/Abstract] OR "Onchocerca volvulus"[MeSH Terms] OR "Onchocerca volvulus"[Title/Abstract]
For Podoconiosis	
#3	"Elephantiasis"[MeSH Terms] OR "Podoconiosis"[Title/Abstract] OR "Mossy foot"[Title/Abstract] OR "Bigfoot Disease"[Title/Abstract] OR ("Elephantiasis"[Title/Abstract] AND ("non filarial"[Title/Abstract] OR "non filarial"[Title/Abstract] OR "Nonfilarial"[Title/Abstract] OR "verrucos*"[Title/Abstract])) OR "Lymphostatic Verrucosis"[Title/Abstract]
For Rabies	
#3	"Rabies"[MeSH Terms] OR "Rabies"[Title/Abstract] OR "Lyssa"[Title/Abstract] OR "Hydrophobia"[Title/Abstract] OR "Rabies virus"[MeSH Terms]
For Scabies and other ectoparasitoses	
#3	"Scabies"[MeSH Terms] OR "Scabies"[Title/Abstract] OR "Sarcoptes

	scabiei"[MeSH Terms] OR ("Sarcoptes"[Title/Abstract] AND ("scabiei"[Title/Abstract] OR "Mange"[Title/Abstract])) OR "seven-year itch"[Title/Abstract]
For Schistosomiasis	
#3	"Schistosomiasis"[MeSH Terms] OR "Schistosomiasis"[Title/Abstract] OR "Bilharziasis"[Title/Abstract] OR "bilharzia"[Title/Abstract] OR ("Fever"[Title/Abstract] AND ("Katayama"[Title/Abstract] OR "snail"[Title/Abstract])) OR "Schistoma"[Title/Abstract] OR "blood flukes"[Title/Abstract] OR "Schistosoma"[Title/Abstract]
For Snakebite envenoming	
#3	"Snake Bites"[MeSH Terms] OR (("Snake Bite"[Title/Abstract] OR "Snakebite"[Title/Abstract] OR "Snake"[Title/Abstract]) AND ("Envenomation"[Title/Abstract] OR "Envenoming"[Title/Abstract]))
For Soil-transmitted helminthiases	
#3	(("soil transmitted"[Title/Abstract] OR "soil transmitted"[Title/Abstract]) AND ("helminths"[Title/Abstract] OR "Helminthiasis"[Title/Abstract])) OR "Ascaris lumbricoides"[MeSH Terms] OR "Ascaris"[Title/Abstract] OR "Trichuris"[MeSH Terms] OR "Trichuris trichiura"[Title/Abstract] OR "hookworm*"[Title/Abstract] OR "Ancylostoma duodenale infection"[Supplementary Concept] OR "Ancylostoma duodenale"[Title/Abstract] OR "Necator americanus"[MeSH Terms] OR "Necator americanus"[Title/Abstract]
For Taeniasis/cysticercosis	
#3	"Taeniasis"[MeSH Terms] OR "Taeniasis"[Title/Abstract] OR "Taenia"[MeSH Terms] OR "Taenia"[Title/Abstract] OR "Cysticercosis"[Title/Abstract] OR

	"Cysticercus"[Title/Abstract] OR "Coenuri"[Title/Abstract] OR "Coenurus"[Title/Abstract] OR "Coenurosis"[Title/Abstract]
For Trachoma	
#3	"Trachoma"[MeSH Terms] OR "Trachoma"[Title/Abstract] OR "Egyptian Ophthalmia"[Title/Abstract] OR "Chlamydia trachomatis"[MeSH Terms] OR "Chlamydia trachomatis"[Title/Abstract]
For Yaws and other endemic treponematoses	
#3	"Yaws"[MH] OR "Yaws"[TIAB] OR "Frambesia"[TIAB] OR "Treponema pallidum"[MH] OR "Treponema pallidum"[TIAB] OR "bejel"[TIAB] OR "pinta"[TIAB] OR "Pinta"[MH] OR "Treponema carateum"[TIAB]
#1 AND #2 AND #3	
