# Supplementary material for: Pharmacological therapies for neglected tropical diseases: a systematic review and evidence gap mapping
Source: Rev Soc Bras Med Trop. 2026 Jul 3;59:e0056-2026. doi: 10.1590/0037-8682-0056-2026 (PMC13331190; doi:10.1590/0037-8682-0056-2026)
Supplement: Supplementary Table 2 [file 1678-9849-rsbmt-59-e0056-2026-md2.pdf]

Supplementary Table 2. Outcome grouping breakdown

Neglected tropical disease	Outcome category	Specific outcomes extracted from included studies
Buruli ulcer	Cure	Healing of lesions without recurrence at 12 months or longer, Complete healing lesions at follow-up, Cure rate
	Healing lesions	Healing time, Changes in lesion size
Chagas disease	Cardiovascular/cerebrovascular parameters	Onset of cardiovascular events, Cardiologic evaluation, Postural response (heart rate, systolic and diastolic arterial blood pressure changes in response to standing), Heart rate changes induced by cough and hyperventilation reflex tests, Dizziness on standing, Number of stress-induced arrhythmias, Hourly extrasystoles with and without paired responses, Non-sustained ventricular tachyarrhythmias, Time to cardiovascular death
	Response to therapy	Return to negative serology values/return to negative values in parasitology testing, Clearance or reduction of anti-T cruzi antibodies, Clinical improvement
	Esophageal function in chagasic achalasia	Esophageal emptying time, Lower esophageal sphincter (LES) basal pressure
	Liver/renal function	Serum bilirubin, serum glutamic pyruvic transaminase, serum glutamic oxalacetic transaminase, creatinina, ureia, glomerular filtration rate
Dengue and Chikungunya	Viremia	Virological log reduction (VLR), Clearance of serum NS1, Cytokine levels, Vaccine efficacy to any and each serotype, Vaccine-specific immune response by neutralizing antibody assay, Geometric mean titles (GMTs) of neutralizing antibodies against DENV-1–4, Proportion of participants seropositive for each of the four DENV serotypes
	Hematological parameters	White blood cell count, Platelet counts, Hemoconcentration, Coagulation
	Fever	Fever clearance time (FCT), Incidence of fever
Dracunculiasis	Healing lesions	Healing time, Changes in lesion size
Echinococcosis	Cure	Clinical cure, parasitological cure, Cyst cure

	Cyst viability	Cyst viability, Inactive cyst, Changes in cyst morphology during and after treatment, Cyst rupture
	Hematological parameters	Levels of leucocytes, Levels of neutrophiles
	Morbidity	Complications, fever, allergic reactions
Foodborne trematodiases	Cure	Clinical cure, Parasitological cure
	Parasitological response	Egg reduction rate, Clearance of Paragonimus eggs from sputum
	Liver function	Serum bilirubin, serum glutamic pyruvic transaminase, serum glutamic oxalacetic transaminase
Human African trypanosomiasis (sleeping sickness)	Cure	Clinical cure, Parasitological cure, Absence of parasites in blood, lymph nodes, and CSF
	Encephalopathic syndrome	Coma type, convulsion type and psychotic reactions, Rate of encephalopathy
Leishmaniasis	Clinical improvement	Improvement of the lesion in terms of size, erythema, inflammation, edema and ulcer re-epithelization
	Cure	Complete clinical response, complete epithelization, complete healing, complete re-epithelization, complete clinical remission
	Treatment failure	Lesions failed to completely heal (100% reepithelialization) by 12 weeks after the start of treatment, No decrease in size or induction, Absence of any change in or worsening of lesions
	Liver function	Serum bilirubin, serum glutamic pyruvic transaminase, serum glutamic oxalacetic transaminase
	Renal toxicity	Nephrotoxicity
Leprosy (Hansen's disease)	Bacillary index	Proportion of M. leprae viable
	Clinical improvement	Disappearance of the lesion, reduction in hypopigmentation/erythema, reduction in the degree of infiltration, reduction in size of the lesion, improvement in cessation in the lesion
	Cure	Complete resolution of the lesion
	Proinflammatory cytokines	Proliferation and IFN-g production
	Serological response	Antibody titles
	Erythema nodoum leprosum	Frequency and severity of erythema nodosum leprosum (ENL), Changes in ENL episode duration
	Liver function	Serum bilirubin, serum glutamic pyruvic transaminase, serum glutamic oxalacetic transaminase

	Neuritis	Pain during the interview or when participants complain of pain in one or more peripheral nerve trunks of the limb(s) during the period of the treatment
	Reversal reactions	Reversal reactions Type 1 and type 2
Lymphatic filariasis	Cure	Complete clearance of microfilaremia and filarial antigemia 12 months after treatment
	Morbidity	Lymphoedema, Adenolymphangitis attacks
	Microfilaremia	Microfilariae levels, microfilaria counts, rate of microfilaremia
	CFA levels	Antigemia, Circuling filarial antigen (CFA) levels
	Filarial dance sing	Presence of adult worm
	Hematological parameters	Complete cell count
	Liver function	Levels of serum creatinine, total bilirubin, aspartate aminotransferase (SGOT), alanine aminotransferase (SGPT), and alkaline phosphatase (AP)
Mycetoma, chromoblastomycosis and other deep mycoses	Healing of lesion	Amelioration of keratotic crusting or scaring of lesions
Onchocerciasis (river blindness)	Microfilaremia	Microfilaria load, Levels of microfilaremia
	Viability and fertility of adult worms	Death of the adult worms
	Visual acuity	Optic disc, chroidoretinal and retinal vasculature
Rabies	Immunogenicity	Rabies virus neutralizing antibody (RVNA) response in geometric mean titers, RVNA titles
	Virus neutralizing antibody	Geometric mean titles of rabies virus neutralized antibody
Scabies and other ectoparasitoses	Cure	Clinical cure, Absence of new lesions and healing of all lesions, Microscopic cure
	Morbidity	Impetigo, eczema
	Pruritus	Improvement in itching
	Improvement of the infection	Presence of signs and symptoms during/after treatment, Incomplete healing of lesions
	Treatment failure	Presence of new lesions after treatment
Schistosomiasis	Cure	Cure rate, Egg reduction, 3 consecutive urine samples without the presence of eggs, Cessation of excretion of viable eggs in stools
	Gut inflammation	Fecal calprotectin
	Immunological response	Cytokine, antibody responses to schistosome worm - SWA, egg SEA antigen, CRP, serum IL-6, serum TNF-alpha
	Intensity of infection	Egg counts

	Morbidity	Urinary tract infection, hematuria, proteinuria, hepatomegaly, splenomegaly
	Liver function	Serum bilirubin, serum glutamic pyruvic transaminase, serum glutamic oxalacetic transaminase
Snakebite	Limb function	Snakebite severity score of all limb functions (pulmonary, cardiovascular, hematologic, renal, nervous system)
	Clotting functions	Clotting time, abnormalities in clotting
Soil-transmitted helminthiases	Antopometric improvement	Weight, height. triceps skinfold. mid- arm circumference, and chest circumference
	Cure	Parasitological cure, Egg counts negative after treatment
	Intensity of infection	Egg counts, egg reduction
	Liver function	Serum bilirubin, serum glutamic pyruvic transaminase, serum glutamic oxalacetic transaminase
Taeniasis/Cysticercosis	Cyst control	Disappearance of cyst, reduction in number of cysts
	Lesion reduction	Lesion disappearance on computed tomography or magnetic resonance imaging
	Morbidity	Presence of gliosis, oedema, raised intracranial, intracranial hypertension, fresh symptoms, disability
Trachoma	Cure	Clinical cure, Parasitological cure, Resolution of clinical sings
	Treatment failure	Progression of the conjunctival inflammation
Yaws and other endemic treponematoses	Cure	Clinical cure, Serological cure, Cure rate, Molecular cure
	Healing lesions	Clinical healing of ulcers, complete or partial healing lesions
