# Supplementary material for: Pharmacological therapies for neglected tropical diseases: a systematic review and evidence gap mapping
Source: Rev Soc Bras Med Trop. 2026 Jul 3;59:e0056-2026. doi: 10.1590/0037-8682-0056-2026 (PMC13331190; doi:10.1590/0037-8682-0056-2026)
Supplement: Supplementary Table 3 [file 1678-9849-rsbmt-59-e0056-2026-md3.pdf]

Supplementary Table 3. Study selection process

Neglected tropical disease	Study design	N studies retrieved	N studies excluded during screening	N reports not retrieved	N studies excluded during eligibility	N included studies
Buruli ulcer	SR	3	0	0	0	**3**
	RCT	10	5	0	0	**5**
	RCT-O	2	-	-	-	**2**
Chagas disease	SR	13	6	0	1	**6**
	RCT	38	20	2	3	**13**
	RCT-O	7	-	-	-	**7**
Dengue and Chikungunya	SR	18	11	1	4	**2**
	RCT	65	25	3	7	**30**
	RCT-O	20	-	-	-	**20**
Dracunculiasis	SR	1	0	0	1	**0**
	RCT	5	0	2	1	**2**
	RCT-O	0	-	-	-	**0**
Echinococcosis	SR	10	3	1	2	**4**
	RCT	27	17	1	2	**7**
	RCT-O	0	-	-	-	**0**
Foodborne trematodiases	SR	0	0	0	0	**0**
	RCT	15	8	1	1	**5**
	RCT-O	1	-	-	-	**1**
Human African trypanosomiasis (sleeping sickness)	SR	8	3	0	2	**3**
	RCT	29	12	0	3	**14**
	RCT-O	2	-	-	-	**2**
Leishmaniasis	SR	54	30	0	11	**13**
	RCT	274	70	22	52	**130**

	RCT-O	11	-	-	-	**11**
Leprosy (Hansen's disease)	SR	31	13	1	13	**4**
	RCT	140	56	8	33	**43**
	RCT-O	4	-	-	-	**4**
Lymphatic filariasis	SR	23	11	0	5	**7**
	RCT	102	24	8	18	**52**
	RCT-O	1	-	-	-	**1**
Mycetoma, chromoblastomycosis and other deep mycoses	SR	3	1	0	1	**1**
	RCT	2	1	0	0	**1**
	RCT-O	0	-	-	-	**0**
Onchocerciasis (river blindness)	SR	5	1	0	3	**1**
	RCT	39	7	5	6	**21**
	RCT-O	4	-	-	-	**4**
Podoconiosis	SR	0	0	0	0	**0**
	RCT	1	0	0	1	**0**
	RCT-O	0	-	-	-	**0**
Rabies	SR	9	2	0	6	**1**
	RCT	75	12	3	46	**14**
	RCT-O	6	-	-	-	**6**
Scabies and other ectoparasitoses	SR	15	8	0	3	**4**
	RCT	80	44	7	2	**27**
	RCT-O	5	-	-	-	**5**
Schistosomiasis	SR	25	14	0	5	**6**
	RCT	119	43	12	21	**43**
	RCT-O	1	-	-	-	**1**
Snakebite	SR	2	0	0	0	**2**
	RCT	24	6	2	4	**12**

	RCT-O	1	-	-	-	**1**
Soil-transmitted helminthiases	SR	14	5	0	4	**5**
	RCT	141	66	10	28	**37**
	RCT-O	3	-	-	-	**3**
Taeniasis/Cysticercosis	SR	16	3	0	6	**7**
	RCT	52	14	2	11	**25**
	RCT-O	1	-	-	-	**1**
Trachoma	SR	24	5	0	14	**5**
	RCT	212	151	3	42	**16**
	RCT-O	0	-	-	-	**0**
Yaws and other endemic treponematoses	SR	2	2	0	0	**0**
	RCT	13	9	0	1	**3**
	RCT-O	1	-	-	-	**1**

Note: N = number; RCT = randomized controlled trial; RCT-O = ongoing randomized controlled trial; SR = systematic review.
