# Supplementary material for: Pharmacological therapies for neglected tropical diseases: a systematic review and evidence gap mapping
Source: Rev Soc Bras Med Trop. 2026 Jul 3;59:e0056-2026. doi: 10.1590/0037-8682-0056-2026 (PMC13331190; doi:10.1590/0037-8682-0056-2026)
Supplement: Supplementary Figures 1–24 [file 1678-9849-rsbmt-59-e0056-2026-md4.pdf]

Supplementary Figure 1 - EGM of Buruli ulcer

Buruli ulcer

INTERVENTIONS	EVIDENCE	OUTCOMES					
		Efficacy			Safety		
		Cure¹	Healing lesions²	Recurrence	Adverse reactions	Treatment discontinuation	Paradoxical reactions
Clarithromycin	SR	2 1	1 1	1 1	2 1		1
	RCT						
	RCT-O						
Clofazimine with or without surgery	SR	2 1	1 1	1 1	2 1		1
	RCT	1	1	1			
	RCT-O						
Ciprofloxacin + Clarithromycin with surgery	SR	1	1	1	1		1
	RCT						
	RCT-O						
Clarithromycin + Ethambutol with or without surgery	SR	2 1	1 1	1 1	2 1		1
	RCT						
	RCT-O						
Dapsone + Rifampicin	SR	2 1	1 1	1 1	2 1		1
	RCT						
	RCT-O						
Rifampicin + Moxifloxacin with surgery	SR	1	1	1	1		1
	RCT						
	RCT-O						
Rifampicin + Streptomycin with or without surgery	SR	2 1	1 1	1 1	2 1		1
	RCT		2 1	1	2 1	1	1
	RCT-O						
Rifampicin + Streptomycin before surgery	SR	2 1	1 1	1 1	2 1		1
	RCT						
	RCT-O						
Rifampicin + Clarithromycin with or without surgery	SR	2 1	1 1	1 1	2 1		1
	RCT		1	1	1	1	1
	RCT-O						
Rifampicin + Ciprofloxacin with or without surgery	SR	2 1	1 1	1 1	2 1		1
	RCT						
	RCT-O						
Rifampicin + Clarithromycin + Ethambutol	SR	2 1	1 1	1 1	2 1		1
	RCT						
	RCT-O						
Rifampicin + Ethambutol + Amikacin	SR	2 1	1 1	1 1	2 1		1
	RCT						
	RCT-O						
Rifampicin-Clarithromycin + Amoxilin-Clavulanate	SR						
	RCT						
	RCT-O	1	1	1	1	1	1
Rifampicin + Streptomycin 2 weeks followed Rifampicin + Clarithromycin 6 weeks	SR	2 1	1 1	1 1	2 1		1
	RCT						
	RCT-O						
Rifampicin + Streptomycin 4 weeks followed Rifampicin + Clarithromycin 4 weeks	SR	2 1	1 1	1 1	2 1		1
	RCT		1		1		
	RCT-O						
Rifampicin + Streptomycin 8 weeks followed surgery after day 28 and 8 more weeks of Rifampicin + Streptomycin	SR	2 1	1 1	1 1	2 1		1
	RCT						
	RCT-O						
Sodium nitrite + Citric acid monohydrate	SR						
	RCT		1		1		
	RCT-O						
Sulfamethoxazole/Trimethoprim	SR	2 1	1 1	1 1	2 1		1
	RCT						
	RCT-O						
Telacebec	SR						
	RCT						
	RCT-O	1	1	1			1

Note: RCT = randomized controlled trial; RCT-O = ongoing randomized controlled trial; SR = systematic review.

¹ Cure = Cure and/or probable cure and/or possible cure;

² Healing lesions = Complete or significative reduction in the surface area of lesion.

Quality assessment of systematic reviews: ■ Critically low; ■ Low; ■ Moderate; ■ High quality.

Risk of bias assessment of RCTs: ■ High; ■ Moderate; ■ Low risk of bias.

Supplementary Figure 2 - EGM of antitrypanosomal drug therapy for Chagas disease

Chagas disease

INTERVENTIONS		EVIDENCE	OUTCOMES: Efficacy – Mortality	Efficacy – Changes in cardiovascular/ cerebrovascular parameters	Efficacy – Response to therapy[1]	Safety – Adverse reactions	Safety – Treatment discontinuation	Safety – Liver/ renal function	Safety – Leucopenia, neutropenia and/or lymphopenia
Antitrypanosomal drug therapy	Allopurinol	SR	1 (Low)		2 (Low)		1 (Low)		1 (Low)
		RCT							
		RCT-O							
	Benznidazole alone or associated to FOS, POS or TA	SR	1 (Low)	2 (Critically low); 1 (Low); 1 (Moderate)	2 (Critically low); 2 (Low); 1 (Moderate)	3 (Critically low); 1 (Low); 1 (Moderate)	2 (Critically low); 1 (Low); 1 (Moderate)	1 (Low)	1 (Low)
		RCT			3 (High); 2 (Low risk of bias)	3 (High); 2 (Low risk of bias)	3 (High); 1 (Low risk of bias)	2 (Low risk of bias)	
		RCT-O		1	5	5	2		
	Fexinidazole	SR							
		RCT			1 (Low risk of bias)	1 (Low risk of bias)			
		RCT-0							
	LXE408	SR							
		RCT							
		RCT-O			1	1	1		
	Nifurtimox	SR	1 (Low)	1 (Low)	1 (Low); 1 (Moderate)	1 (Low); 1 (Moderate)	1 (Moderate)		
		RCT							
		RCT-O							
	Posaconazole	SR		1 (Low)	1 (Low)	1 (Moderate)		1 (Low)	
		RCT			1 (High)	1 (High)	1 (High)		
		RCT-O							
	Ravuconazole	SR		1 (Low)	1 (Low)	1 (Moderate)	1 (Moderate)	1 (Low)	
		RCT			1 (Low risk of bias)	1 (Low risk of bias)		1 (Low risk of bias)	
		RCT-O							

Note: RCT = randomized controlled trial; RCT-O = ongoing randomized controlled trial; SR = systematic review; FOS = fosravuconazol; POS = posaconazole; TA = thioctic acid.

[1] Response to therapy = return to negative serology values/return to negative values in parasitology testing.

The number inside the boxes indicate the number of studies assessing each drug/outcome.

Quality assessment of systematic reviews: ■ Critically low; ■ Low; ■ Moderate; ■ High quality.

Risk of bias assessment of RCTs: ■ High; ■ Moderate; ■ Low risk of bias.

Supplementary Figure 3 - EGM of symptomatic therapy for Chagas disease

Chagas disease

	INTERVENTIONS	EVIDENCE	OUTCOMES					
			Efficacy				Safety	
			Mortality	Hospitalization	Changes in cardiovascular/ cerebrovascular parameters	Esophageal function in chagasic achalasia	Adverse reactions	Liver/ renal function
Symptomatic therapy	Amiodarone hydrochloride	SR						
		RCT			1		1	
		RCT-O	1	1	1			
	Botulinum toxin injection (in in the LES)	SR						
		RCT				1	1	
		RCT-O						
	Cronassial (mixed gangliosides)	SR						
		RCT			1		1	
		RCT-O						
	Disopyramide	SR						
		RCT			1		1	
		RCT-O						
	Enalapril	SR						
		RCT						
		RCT-O			1			
	Isosorbide dinitrate	SR						
		RCT				1	1	
		RCT-O						
	Ivabradine	SR						
		RCT	1	1	1		1	
		RCT-O						
	Nifedipine	SR						
		RCT				1	1	
		RCT-O						
	Pyridostigmine	SR						
		RCT			1		1	
		RCT-O						
	Sacubitril/ valsartan	SR						
		RCT						
		RCT-O			1			
	Sotalol	SR						
		RCT			1		1	1
		RCT-O						

Note: RCT = randomized controlled trial; RCT-O = ongoing randomized controlled trial; SR = systematic review; LES = lower esophageal sphincter.
The number inside the boxes indicate the number of studies assessing each drug/outcome.
Quality assessment of systematic reviews: ■ Critically low; ■ Low; ■ Moderate; ■ High quality.
Risk of bias assessment of RCTs: ■ High; ■ Moderate; ■ Low risk of bias.

Supplementary Figure 4 - EGM of symptomatic therapy for Dengue/Chikungunya

Dengue and Chikungunya

INTERVENTIONS		EVIDENCE	OUTCOMES						
			Efficacy			Safety			
			Mortality	Hospitalization	Viremia[1]	Fever	Hematological parameters[2]	Adverse reactions	Intensity of pain
Symptomatic interventions	Anakinra	SR							
		RCT							
		RCT-O	1	1	1	1	1	1	
	Balapiravir	SR							
		RCT			1			1	
		RCT-O							
	Chloroquine	SR			1	1	1		
		RCT			1	1; 1	1	1; 1	1
		RCT-O							
	Celgosivir	SR							
		RCT			1	1	1	1	
		RCT-O							
	Dexamethasone	SR							
		RCT							
		RCT-O	1	1					
	Doxycycline	SR							
		RCT	1		1				
		RCT-O							
	Fenretinide	SR							
		RCT							
		RCT-O			1	1		1	1
	Hydrocortisone	SR							
		RCT	1			1			
		RCT-O							
	Hydroxychloro-quine	SR			1				
		RCT							
		RCT-O							
	Lovastatin	SR							
		RCT	1		1	1		1	
		RCT-O							
	Melatonin	SR							
		RCT							
		RCT-O				1	1		
	Molnupiravir	SR							
		RCT							
		RCT-O			1	1	1		
	Paracetamol	SR							
		RCT	1	1	1	1			1
		RCT-O							
	Prednisolone	SR							
		RCT			2	1		1	
		RCT-O							
	Recombinant activated factor VII	SR							
		RCT					1	1	
		RCT-O							
	Zanamivir	SR							
		RCT							
		RCT-O						1	
	Hydroxychloroquine + Aceclofenac	SR			1				
		RCT							
		RCT-O							
	Hydroxychloroquine + Methotrexate	SR			1				
		RCT							
		RCT-O							
	Hydroxychloroquine + Methotrexate + Sulfasazine	SR			1				
		RCT							
		RCT-O							

Note: RCT = randomized controlled trial; RCT-O = ongoing randomized controlled trial; SR = systematic review.
[1] Viremia = Reductions in viral load (VLR and NS1 clearance), cytokine levels, vaccine efficacy, and neutralizing antibody response (GMTs and seropositivity) across DENV-1–4 serotypes;
[2] Hematological parameters = Evaluation of white blood cell and platelet counts, hemoconcentration levels, and coagulation profile.
The number inside the boxes indicate the number of studies assessing each drug/outcome.
Quality assessment of systematic reviews: ■ Critically low; ■ Low; ■ Moderate; ■ High quality.
Risk of bias assessment of RCTs: ■ High; ■ Moderate; ■ Low risk of bias.

Supplementary Figure 5 - EGM of vaccines for Dengue/Chikungunya

Dengue and Chikungunya

INTERVENTIONS		EVIDENCE	OUTCOMES						
			Efficacy			Safety			
			Mortality	Hospitalization	Viremia[1]	Fever	Hematological parameters[2]	Adverse reactions	Intensity of pain
Vaccines	BBV87 chikungunya vaccine	SR							
		RCT							
		RCT-O			1			1	
	JNJ-64281802 dengue vaccine	SR							
		RCT							
		RCT-O			1			1	
	Lipid nanoparticle mRNA	SR							
		RCT						1	
		RCT-O							
	EYU688	SR							
		RCT							
		RCT-O			1	1	1	1	
	rDEN1D30 dengue vaccine	SR							
		RCT			1			1	
		RCT-O			1			1	
	rDEN4Δ30-200,201 dengue vaccine	SR							
		RCT			1		1	1	
		RCT-O							
	rDEN2Δ30-7169 dengue vaccine	SR							
		RCT							
		RCT-O			1			1	
	PepGNP-Dengue	SR							
		RCT			1			1	
		RCT-O							
	Tetravalent dengue vaccine	SR							
		RCT		1	13 1			13 1	
		RCT-O		1	3	1		3	
	TDVH4 dengue vaccine	SR							
		RCT			1			1	
		RCT-O			3			3	
	V181 dengue vaccine	SR							
		RCT							
		RCT-O			1			1	
	VLA1553 chikungunya vaccine	SR							
		RCT							
		RCT-O			2			2	
	VRC-CHKVLP059-00-VP chikungunya vaccine	SR							
		RCT			1			1	
		RCT-O							

Note: RCT = randomized controlled trial; RCT-O = ongoing randomized controlled trial; SR = systematic review.

[1] Viremia = Reductions in viral load (VLR and NS1 clearance), cytokine levels, vaccine efficacy, and neutralizing antibody response (GMTs and seropositivity) across DENV-1–4 serotypes;

[2] Hematological parameters = Evaluation of white blood cell and platelet counts, hemoconcentration levels, and coagulation profile.

The number inside the boxes indicate the number of studies assessing each drug/outcome.

Quality assessment of systematic reviews: ■ Critically low; ■ Low; ■ Moderate; ■ High quality.

Risk of bias assessment of RCTs: ■ High; ■ Moderate; ■ Low risk of bias.

Supplementary Figure 6 - EGM of Dracunculiasis

Dracunculiasis

INTERVENTIONS	EVIDENCE	OUTCOMES		
		Efficacy		Safety
		Healing lesions[1]	Extrusion of worms	Adverse reactions
Niridazole	SR			
	RCT	1	1	1
	RCT-O			
Niridazole + Tetanus toxoid injection	SR			
	RCT	1		
	RCT-O			

Note: RCT = randomized controlled trial; RCT-O = ongoing randomized controlled trial; SR = systematic review.

[1] Healing lesions = Measurement of total healing time and longitudinal changes in lesion size.

The number inside the boxes indicate the number of studies assessing each drug/outcome.

Quality assessment of systematic reviews: ■ Critically low; ■ Low; ■ Moderate; ■ High quality.

Risk of bias assessment of RCTs: ■ High; ■ Moderate; ■ Low risk of bias.

Supplementary Figure 7 - EGM of Echinococcosis

Echinococcosis

INTERVENTIONS	EVIDENCE	OUTCOMES								
		Efficacy							Safety	
		Mortality	Cure[1]	Hospitalization	Cyst viability[2]	Calcification of the cyst	Morbidity	Recurrence	Adverse reactions	Hematological parameters[3]	Assessment of transaminases (ALT and AST)
Albendazole with or without surgery	SR	1, 1	1	1	2, 1		1	2, 1			
	RCT		2		1	2		1	2	1	1
	RCT-O										
Albendazole with catheterization	SR	1		1	1			1			
	RCT										
	RCT-O										
Albendazole + PAIR	SR	1, 1	1	1, 1	1		1	1, 1			
	RCT		1	1				1	1		
	RCT-O										
Albendazole + percutaneous drainage	SR										
	RCT				1						
	RCT-O										
Albendazole + Praziquantel with or without surgery	SR	1	1		2		1	2			
	RCT				1						
	RCT-O										
Mebendazole	SR	1	1		2		1	2			
	RCT		1			2		1	1		
	RCT-O										
Mebendazole + PAIR	SR	1	1	1			1	1			
	RCT										
	RCT-O										

Note: RCT = randomized controlled trial; RCT-O = ongoing randomized controlled trial; SR = systematic review.
[1] Cure = Clinical cure; parasitological and/or cyst cure;
[2] Cyst viability = Assessment of cyst status, morphological changes during and after treatment, and occurrence of cyst rupture;
[3] Morbidity = Incidence of clinical complications, presence of fever, and occurrence of allergic reactions;
[3] Hematological parameters = Measurement of leucocyte and neutrophil levels.
The number inside the boxes indicate the number of studies assessing each drug/outcome.
Quality assessment of systematic reviews: ■ Critically low; ■ Low; ■ Moderate; ■ High quality.
Risk of bias assessment of RCTs: ■ High; ■ Moderate; ■ Low risk of bias.

Supplementary Figure 8 - EGM of Foodborne trematodiases

Foodborne trematodiases

INTERVENTIONS	EVIDENCE	OUTCOMES				
		Efficacy			Safety	
		Cure[1]	Parasitological response[2]	Radiological signs	Adverse reactions	Liver function[3]
Artesunate	SR					
	RCT	1	1		1	
	RCT-O					
Mefloquine	SR					
	RCT	1	1		1	
	RCT-O					
Mefloquine-Artesunate	SR					
	RCT	1	1		1	
	RCT-O					
Oxfendazole	SR					
	RCT					
	RCT-O	1	1		1	
Praziquantel	SR					
	RCT	3	2		3	
	RCT-O					
Tribendimidine	SR					
	RCT	2	2		2	
	RCT-O					
Triclabendazole	SR					
	RCT	1	1	1	1	1
	RCT-O					

Note: RCT = randomized controlled trial; RCT-O = ongoing randomized controlled trial; SR = systematic review.

[1] Cure = clinical cure and/or parasitological cure;

[2] Parasitological cure = Egg reduction rate and total clearance of Paragonimus eggs from sputum;

[3] Liver function = Measurement of serum bilirubin, glutamic pyruvic transaminase (SGPT/ALT), and glutamic oxalacetic transaminase (SGOT/AST).

The number inside the boxes indicate the number of studies assessing each drug/outcome.

Quality assessment of systematic reviews: ■ Critically low; ■ Low; ■ Moderate; ■ High quality.

Risk of bias assessment of RCTs: ■ High; ■ Moderate; ■ Low risk of bias.

Supplementary Figure 9 - EGM of Human African trypanosomiasis (sleeping sickness)

Human African trypanosomiasis

INTERVENTIONS	EVIDENCE	OUTCOMES						
		Efficacy					Safety	
		Cure[1]	Recurrence	Encephalopathic syndrome[2]	Mortality	Cerebrospinal fluid white blood cells counts	Adverse reactions	Death attributed to treatment
Acoziborole	SR							
	RCT							
	RCT-O	1					2	1
Eflornithine	SR	1 1	1				1 1	1
	RCT	1 2	2 2		2 1		2 2	
	RCT-O							
Fexinidazole	SR	1 1	1				1 1	
	RCT	1					1	
	RCT-O							
Melarsoprol	SR	1 1	1				1 1	1
	RCT	1	2	3		1	4	4
	RCT-O							
Nifurtimox	SR	1	1		1		1	
	RCT		1				1	1
	RCT-O							
Pafuramidine	SR							
	RCT	2	2		1		2	
	RCT-O							
Pentamidine	SR	1	1				1	1
	RCT	2	2		1		2	
	RCT-O							
Melarsoprol + Eflornithine	SR	1	1				1	1
	RCT	1	1				1	1
	RCT-O							
Melarsoprol + Nifurtimox	SR	1	1				1	1
	RCT	1	2				2	2
	RCT-O							
Melarsoprol + Prednisolone	SR	1	1				1	1
	RCT			1		1		1
	RCT-O							
Nifurtimox- eflornithine	SR	1 2	2		1		1 2	
	RCT	3 2	2 2		1		3 2	1
	RCT-O							

Note: RCT = randomized controlled trial; RCT-O = ongoing randomized controlled trial; SR = systematic review.

[1] Cure = Clinical and parasitological cure, characterized by the absence of parasites in blood, lymph nodes, and cerebrospinal fluid (CSF);

[2] Encephalopathic syndrome = Classification of coma and convulsion types, occurrence of psychotic reactions, and the overall rate of encephalopathy.

The number inside the boxes indicate the number of studies assessing each drug/outcome.

Quality assessment of systematic reviews: ■ Critically low; ■ Low; ■ Moderate; ■ High quality.

Risk of bias assessment of RCTs: ■ High; ■ Moderate; ■ Low risk of bias.

Supplementary Figure 10 - EGM of Cutaneous leishmaniasis

Cutaneous leishmaniasis

INTERVENTIONS	EVIDENCE	OUTCOMES: Efficacy – Clinical improvement[1]	Efficacy – Cure[2]	Efficacy – Mortality	Efficacy – Quality of life	Efficacy – Recurrence	Efficacy – Treatment failure[3]	Safety – Adverse reactions	Safety – Liver function	Safety – Nausea	Safety – Renal toxicity	Safety – Treatment discontinuation
18-Methoxycoronaridine	SR											
	RCT											
	RCT-O		1				1					
Allopurinol	SR		2, 2	1	2	1	1	2				
	RCT	2	2				2	1				
	RCT-O											
Aminosidine	SR		1, 1	1	1	1		1				
	RCT	1	2, 1			1	2	2	1			
	RCT-O											
Amphotericin B	SR		3, 3	1	1	2		3				
	RCT	1, 1	1, 1			2	1	1, 1				
	RCT-O	1	1					2				
Azithromycin	SR		1, 2	1	2	1		2				
	RCT	1	4			1	3	3				
	RCT-O											
Chloroquine	SR											
	RCT		2									
	RCT-O											
Ciprofloxacin	SR											
	RCT	1										
	RCT-O											
Clotrimazole	SR		1									
	RCT											
	RCT-O											
Dapsone	SR		2, 2		1			2				
	RCT		1							1		
	RCT-O											
Doxycycline	SR											
	RCT		1									
	RCT-O	1										
Fluconazole	SR		1, 3	1	2	1		3				
	RCT											
	RCT-O											
Imiquimod	SR		1, 2	1	1	1	1	2				
	RCT	1	1			1	1	1				
	RCT-O											
Itraconazole	SR		2, 2		1			2				
	RCT	3	1, 1				1	1	1		1	
	RCT-O											
Ketoconazole	SR		2, 3	1	2	1		3				
	RCT	1, 1	2				2	1, 1				
	RCT-O											
Meglumine Antimoniate	SR	1	4, 3	1	2	1	1	3				
	RCT	21, 9	31, 13			11, 4	23, 9	21, 9	1			2
	RCT-O	2	3				1	3				
Metronidazole	SR											
	RCT		1			1		1				
	RCT-O											
Miconazole	SR		1		1			1				
	RCT											
	RCT-O											
Miltefosine	SR		2	1	2	1		2				
	RCT	3, 1	5, 2			2, 1	3	6, 2				1
	RCT-O	2	2					3				
Nitric oxide	SR											
	RCT	1				1	1	1				
	RCT-O											
Paromomycin	SR	1	4, 2	1	2	1, 1	1	1, 2				
	RCT	3, 3	4, 4			2, 3	3, 1	1, 3			2	
	RCT-O											

Cutaneous leishmaniasis (continued)

INTERVENTIONS	EVIDENCE	OUTCOMES										
		Efficacy						Safety				
		Clinical improvement[1]	Cure[2]	Mortality	Quality of life	Recurrence	Treatment failure[3]	Adverse reactions	Liver function	Nausea	Renal toxicity	Treatment discontinuation
Pentamidine	SR		3 2	1	1	1	1	2				
	RCT	4	6			2	4	4	1			
	RCT-O											
Rifampicin	SR		2 1		1			1				
	RCT											
	RCT-O											
Sodium stibogluconate	SR		4 3	1	2	1	1	3				
	RCT	4 4	3 6 1			1 3 1	3 3	3 6 1	1			
	RCT-O	1										
Trichloroacetic acid	SR		2									
	RCT	1	1				1					
	RCT-O											
Voriconazole	SR											
	RCT											
	RCT-O	1										
Zinc sulphate	SR		2 1		1			1				
	RCT		2					1				
	RCT-O											
5-aminolaevulinic acid hydrochloride + Topical photodynamic therapy	SR											
	RCT	1	1				1					
	RCT-O											
Amphotericin B + Itraconazole	SR		1									
	RCT	1				1		1				
	RCT-O											
Artesunate + Sulphamethoxypyridazine/pyrimethamine	SR		1		1			1				
	RCT											
	RCT-O											
Azithromycin + Allopurinol	SR		1		1			1				
	RCT	1	1				1	1				
	RCT-O											
LEISH-F1 + MPL-SE vaccine	SR											
	RCT					1		1				
	RCT-O											
Meglumine antimoniate + Allopurinol	SR		2 1	1	1	1		1				
	RCT	3	3				3	1				
	RCT-O											
Meglumine antimoniate + Cimetidine	SR											
	RCT	1	1				1	1				
	RCT-O											
Meglumine antimoniate + CO2 laser	SR		1									
	RCT											
	RCT-O											
Meglumine antimoniate + Cryotherapy	SR		2									
	RCT	1	2			1	1	1				
	RCT-O											
Meglumine antimoniate + Diphencyprone	SR											
	RCT	1										
	RCT-O											
Meglumine antimoniate + honey	SR		1									
	RCT											
	RCT-O											
Meglumine antimoniate + IFNγ	SR		1 1		1			1				
	RCT	1 1	1					1 1				
	RCT-O											
Meglumine antimoniate + Imiquimod	SR		1 2	1	2	1		2				
	RCT	2 1	2 1			2	1 1	1 1				
	RCT-O											
Meglumine antimoniate + Itraconazole	SR											
	RCT	1	1				1					
	RCT-O											

Cutaneous leishmaniasis (continued)

INTERVENTIONS	EVIDENCE	OUTCOMES										
		Efficacy						Safety				
		Clinical improvement[1]	Cure[2]	Mortality	Quality of life	Recurrence	Treatment failure[3]	Adverse reactions	Liver function	Nausea	Renal toxicity	Treatment discontinuation
Meglumine antimoniate + Lidocaine	SR		1									
	RCT											
	RCT-O											
Meglumine antimoniate + Omeprazole	SR											
	RCT		1									
	RCT-O											
Meglumine antimoniate + Paromomycin	SR		2	1	2	1		2				
	RCT											
	RCT-O											
Meglumine antimoniate + Paromomycin + Bethylbenzethonium chloride	SR											
	RCT		1			1	1					
	RCT-O											
Meglumine antimoniate + Pentoxifylline	SR		2, 1	1	1	1		1				
	RCT	1	4			1	2	2				
	RCT-O		1			1	1					
Meglumine antimoniate + Tamoxifen	SR		1	1	1	1		1				
	RCT		1					1				
	RCT-O											
Meglumine antimoniate + Tofacitinib	SR											
	RCT											
	RCT-O		1					1				
Meglumine antimoniate + TCA	SR		1									
	RCT											
	RCT-O											
Meglumine antimoniate + Zinc sulphate	SR		1	1	1	1		1				
	RCT											
	RCT-O											
Meglumine antimoniate + GM-CSF	SR											
	RCT		1				1	1				
	RCT-O											
Miltefosine + GM-CSF	SR											
	RCT		1					1				
	RCT-O											
Miltefosine + Pentoxifylline	SR											
	RCT											
	RCT-O		2			1	1	1				
Miltefosine + Thermotherapy	SR											
	RCT											
	RCT-O	1	1					1				
Nitric oxide + Cryotherapy	SR											
	RCT	1	1				1					
	RCT-O											
Paromomycin + Methylbenzethonium chloride	SR	1	1, 2	1	2	1, 1		1, 2				
	RCT	1	3				3	1				
	RCT-O											
Paromomycin + Gentamicin	SR	1	1, 1	1	1	1, 1		1, 1				
	RCT	2	1, 4			4		1, 3			2	
	RCT-O											
Paromomycin + Urea	SR											
	RCT	1	2				1	1				
	RCT-O											
Rifampicin + Omeprazole	SR											
	RCT		1				1					
	RCT-O											
Sodium Stibogluconate + CO2 laser	SR											
	RCT	1						1				
	RCT-O											
Sodium stibogluconate + Allopurinol	SR		1	1	1	1		1				
	RCT	3	2			1	2	1				
	RCT-O											

Cutaneous leishmaniasis (continued)

INTERVENTIONS	EVIDENCE	OUTCOMES										
		Efficacy						Safety				
		Clinical improvement[1]	Cure[2]	Mortality	Quality of life	Recurrence	Treatment failure[3]	Adverse reactions	Liver function	Nausea	Renal toxicity	Treatment discontinuation
Sodium stibogluconate + Cryotherapy	SR		2									
	RCT											
	RCT-O											
Sodium stibogluconate + Dapsone	SR		1									
	RCT											
	RCT-O											
Sodium stibogluconate + Imiquimod	SR											
	RCT	1	1				1	1				
	RCT-O											
Sodium stibogluconate + Ketoconazole	SR		1		1			1				
	RCT		1				1	1				
	RCT-O											
Sodium stibogluconate + Pentoxifylline	SR		1	1	1	1		1				
	RCT											
	RCT-O											
Sodium stibogluconate + Rifampicin	SR		1									
	RCT											
	RCT-O											
Sodium stibogluconate + Zinc sulphate	SR		1		1			1				
	RCT											
	RCT-O											
Terbinafine + Cryotherapy	SR		1		1			1				
	RCT											
	RCT-O											
Tioxolone + Benzoxonium chloride + cryotherapy	SR											
	RCT		1			1		1				
	RCT-O											
TCA + Radiofrequency head therapy	SR		1									
	RCT											
	RCT-O											

Note: RCT = randomized controlled trial; RCT-O = ongoing randomized controlled trial; SR = systematic review.
[1] Clinical improvement = Improvement of the lesion in terms of size, erythema, inflammation, edema and ulcer re-epithelization;
[2] Cure = Complete clinical response, complete epithelization, complete healing, complete re-epithelization or complete clinical remission;
[3] Treatment failure = Absence of lesion reduction, persistence of clinical signs (erythema, edema, or inflammation), or worsening of the ulcerated area after the intervention.
The number inside the boxes indicate the number of studies assessing each drug/outcome.
Quality assessment of systematic reviews: ■ Critically low; ■ Low; ■ Moderate; ■ High quality.
Risk of bias assessment of RCTs: ■ High; ■ Moderate; ■ Low risk of bias.

Supplementary Figure 11 - EGM of Visceral leishmaniasis

Visceral leishmaniasis

INTERVENTIONS	EVIDENCE	OUTCOMES: Efficacy – Clinical improvement[1]	Efficacy – Cure[2]	Efficacy – Mortality	Efficacy – Recurrence	Efficacy – Treatment failure[3]	Safety – Adverse reactions	Safety – Fever	Safety – Liver, spleen or renal function	Safety – Nephrotoxicity	Safety – Ototoxicity	Safety – Treatment discontinuation
Allopurinol	SR			1			1					
	RCT											
	RCT-O											
Aminosidine	SR		1	1			1					
	RCT											
	RCT-O											
Amphotericin B	SR		2	2			2					
	RCT	4	15, 1		8, 1	4, 1	12, 1	1	2	1	1	
	RCT-O		1	1								
Atovaquone	SR			1			1					
	RCT											
	RCT-O											
Fluconazole	SR			1			1					
	RCT											
	RCT-O											
Ivermectin	SR											
	RCT											
	RCT-O	1					1					
Ketoconazole	SR			1			1					
	RCT											
	RCT-O											
LXE408	SR											
	RCT											
	RCT-O		2	2								
Meglumine antimoniate	SR		1	1			1					
	RCT	1	2		2	2	1					
	RCT-O											
Methylglucamine antimoniate	SR											
	RCT	1	1		1		1					
	RCT-O											
Metronidazole	SR			1			1					
	RCT											
	RCT-O											
Miltefosine	SR		2	2			2					
	RCT		3		2	1	3					1
	RCT-O	1					1					
Paromomycin	SR		2	2			2					
	RCT		5		1	2	5	1	1	1	1	
	RCT-O											
Pentamidine	SR		2	2			2					
	RCT											
	RCT-O											
Roxithromycin	SR			1			1					
	RCT											
	RCT-O											
Sitamaquine	SR		1	2			2					
	RCT		1				1					
	RCT-O											
Sodium stibogluconate	SR		1	2			2					
	RCT	1	7		1		5	3	2			1
	RCT-O											
Amphotericin B + Miltefosine	SR		1	2			2					
	RCT		6	1	1		6					2
	RCT-O											
Amphotericin B + Paromomycin	SR		1	2			2					
	RCT		2	1			2					
	RCT-O											
Amphotericin B + Sodium stibogluconate	SR											
	RCT		1				1					
	RCT-O											
Atovaquone + Fluconazole	SR			1			1					
	RCT											
	RCT-O											

Visceral leishmaniasis (continued)

INTERVENTIONS	EVIDENCE	OUTCOMES										
		Efficacy					Safety					
		Clinical improvement[1]	Cure[2]	Mortality	Recurrence	Treatment failure[3]	Adverse reactions	Fever	Liver, spleen or renal function	Nephrotoxicity	Ototoxicity	Treatment discontinuation
Pentavalent antimony + GM-CSF	SR											
	RCT						1					
	RCT-O											
Meglumine antimoniate + Amphotericin B	SR											
	RCT	1	1		1	1	1					
	RCT-O											
Paromomycin + Miltefosine	SR		1	2			2					
	RCT		4	1			4					1
	RCT-O											
Pentamidine + Allopurinol	SR			1			1					
	RCT											
	RCT-O											
Sodium stibogluconate + Aminosidine	SR			1			1					
	RCT		2		1		2					
	RCT-O											
Sodium stibogluconate + Paromomycin	SR			1			1					
	RCT		2			1	2					
	RCT-O		1	1								

Note: RCT = randomized controlled trial; RCT-O = ongoing randomized controlled trial; SR = systematic review.
[1] Clinical improvement = Improvement of the lesion in terms of size, erythema, inflammation, edema and ulcer re-epithelization;
[2] Cure = Complete clinical response, complete epithelization, complete healing, complete re-epithelization or complete clinical remission;
[3] Treatment failure = Absence of lesion reduction, persistence of clinical signs (erythema, edema, or inflammation), or worsening of the ulcerated area after the intervention.
The number inside the boxes indicate the number of studies assessing each drug/outcome.
Quality assessment of systematic reviews: ■ Critically low; ■ Low; ■ Moderate; ■ High quality.
Risk of bias assessment of RCTs: ■ High; ■ Moderate; ■ Low risk of bias.

Supplementary Figure 12 - EGM of Leprosy (Hansen's disease)

Leprosy																	
INTERVENTIONS	EVIDENCE	OUTCOMES															
		Efficacy								Safety							
		Bacillary index[1]	Clinical improvement[2]	Cure[3]	Improvement in nerve function	Proinflammatory cytokines[4]	Proportion of M. leprae viable	Recurrence	Serological response[5]	Adverse reactions	Antibiotic resistance	Erythema nodoum leprosum[6]	Hepatotoxicity	Liver function[7]	Neuritis[8]	Reversal reactions[9]	Treatment discontinuation
Amitriptyline	SR																
	RCT																
	RCT-O		1							1							
CC-11050	SR																
	RCT																
	RCT-O	1	1			1						1					
Clarithromycin	SR																
	RCT	1					1			1							
	RCT-O																
Clofazimine	SR			1						1							
	RCT						1					1					
	RCT-O																
Dapsone	SR			1						1							
	RCT	1						1			1						
	RCT-O																
Ethionamide	SR																
	RCT	1	1				1			1			1				
	RCT-O																
Etisul (diethyl dithioisophthalate)	SR			1						1							
	RCT																
	RCT-O																
Indomethacin	SR			1						1							
	RCT																
	RCT-O																
Metformin	SR																
	RCT																
	RCT-O				1					1						1	
Minocycline	SR																
	RCT	1					1			1							
	RCT-O																
Mycobacterium vaccine	SR																
	RCT	1						1		1							
	RCT-O																
Ofloxacin	SR	1	1	2				1		2					1		
	RCT	2					2			1							
	RCT-O																
Pefloxacin	SR																
	RCT	1					1										
	RCT-O																
Pentoxifylline	SR			1						1							
	RCT		1							1							
	RCT-O																
Phenytoin sodium-NaCl suspension	SR																
	RCT		1							1							
	RCT-O																
Prednisolone	SR			1						1							
	RCT	1	1		1, 2			1, 1, 1		3, 2					1	1, 1	
	RCT-O									1		1					
Protionamide	SR																
	RCT	1	1				1			1			1				
	RCT-O																
Regular insulin	SR																
	RCT		1	1													
	RCT-O																

Leprosy (continued)

INTERVENTIONS	EVIDENCE	OUTCOMES															
		Efficacy								Safety							
		Bacillary index[1]	Clinical improvement[2]	Cure[3]	Improvement in nerve function	Proinflammatory cytokines[4]	Proportion of M. leprae viable	Recurrence	Serological response[5]	Adverse reactions	Antibiotic resistance	Erythema nodoum leprosum[6]	Hepatoxicity	Liver function[7]	Neuritis[8]	Reversal reactions[9]	Treatment discontinuation
Rifampicin	SR	1	1	2				1		2					1		
	RCT	1		1			1	1	1	1							
	RCT-O																
Thalidomide	SR			1						1							
	RCT	1	2					1		2							
	RCT-O																
Thiosemicarbazone	SR			1						1							
	RCT																
	RCT-O																
Clarithromycin + Clofazimine	SR			1				1		1							
	RCT																
	RCT-O																
Clarithromycin + Minocycline	SR			1				1		1							
	RCT	2					2			2							
	RCT-O																
Clarithromycin + Rifampicin + Ofloxacin	SR			1				1		1							
	RCT																
	RCT-O																
Clarithromycin + Rifampicin + Sparfloxacin + Minocycline	SR			1				1		1							
	RCT																
	RCT-O																
Clofazimine + Dapsone + Ofloxacin	SR			1						1							
	RCT	1	1				2			2				1			
	RCT-O																
Clofazimine + Dapsone + Pheniramine maleate	SR																
	RCT	1	1														
	RCT-O																
Clofazimine + Prednisolone	SR			1						1							
	RCT																
	RCT-O																
Dapsone + Clofazimine	SR			1						1							
	RCT	2	2				2			2				1			
	RCT-O																
Dapsone + Nicotinamide	SR			1						1							
	RCT																
	RCT-O																
Dapsone + Protionamide + Rifampicin	SR																
	RCT									1			1				1
	RCT-O																
Dapsone + Rifampicin + Isoniazid + Protionamide	SR																
	RCT	1						1			1						
	RCT-O																
Dapsone + Thiosemicarbazone	SR			1						1							
	RCT																
	RCT-O																
Isonizid + Rifampicin	SR																
	RCT									1			1				1
	RCT-O																
Isoniazid + Thioacetazone	SR			1						1							
	RCT																
	RCT-O																

Leprosy (continued)

INTERVENTIONS	EVIDENCE	OUTCOMES															
		Efficacy								Safety							
		Bacillary index[1]	Clinical improvement[2]	Cure[3]	Improvement in nerve function	Proinflammatory cytokines[4]	Proportion of M. leprae viable	Recurrence	Serological response[5]	Adverse reactions	Antibiotic resistance	Erythema nodoum leprosum[6]	Hepatoxicity	Liver function[7]	Neuritis[8]	Reversal reactions[9]	Treatment discontinuation
MDT	SR	1	1	2 1				1 1		2 1					1		
	RCT	8	3			2		3 1	3	6 1			1	2		2	1
	RCT-O																
MDT + Acedapsone	SR			1						1							
	RCT																
	RCT-O	1	1	1				1		1					1		
MDT + Clofazimine	SR																
	RCT	1					1			1							
	RCT-O																
MDT + Ethionamide	SR																
	RCT			1				1									
	RCT-O																
MDT + Isoniazid	SR			1						1							
	RCT	1	1														
	RCT-O																
MDT + Mycobacterium vaccine	SR			1						1							
	RCT																
	RCT-O																
MDT + Ofloxacin	SR			1						1							
	RCT							1		1							
	RCT-O	1	1	1				1		1					1		
MDT + Pefloxacin	SR			1						1							
	RCT																
	RCT-O																
MDT + Prednisolone	SR			1						1							
	RCT																
	RCT-O																
MDT + Recombinant human (rh) IFN-g/m2	SR																
	RCT	2				2			2								
	RCT-O																
Minocycline + Clarithromycin + Ofloxacin	SR			1				1		1							
	RCT	1					1			1							
	RCT-O																
Prednisolone + Azathioprine	SR			1						1							
	RCT									1					1		
	RCT-O																
Prednisolone + Ciclosporin	SR			1						1							
	RCT				1			1		1							
	RCT-O																
Prednisolone + Methotrexate	SR																
	RCT																
	RCT-O									1		1					
Prednisolone + Methylprednisolone IV +	SR																
	RCT				1					1							
	RCT-O																
Prednisolone + Pentoxifylline	SR			1						1							
	RCT																
	RCT-O																
Rifampicin + Clofazimine	SR			1				1		1							
	RCT			1				1									
	RCT-O																

Leprosy (continued)

INTERVENTIONS	EVIDENCE	OUTCOMES															
		Efficacy								Safety							
		Bacillary index[1]	Clinical improvement[2]	Cure[3]	Improvement in nerve function	Proinflammatory cytokines[4]	Proportion of M. leprae viable	Recurrence	Serological response[5]	Adverse reactions	Antibiotic resistance	Erythema nodoum leprosum[6]	Hepatoxicity	Liver function[7]	Neuritis[8]	Reversal reactions[9]	Treatment discontinuation
Rifampicin + Dapsone	SR	1	1	2 1				1 1		2 1					1		
	RCT	2 1	1 1	1				2 2	1	4 1	2				1	1	1 1
	RCT-O																
Rifampicin + Isoproduran	SR			1						1							
	RCT																
	RCT-O																
Rifampicin + Ofloxacin	SR			1						1							
	RCT								1								
	RCT-O																
Rifampicin + Sparfloxacin + Clarithromycin + Minocycline	SR	1	1	1				1		1					1		
	RCT	1	1							1						1	
	RCT-O																
ROM	SR	1	2	2 1				1 1		3 1					1		
	RCT		1 3	1 2				1 3		1 2					1	2	1
	RCT-O																
ROM + Clarithromycin	SR			1 1				1		1 1							
	RCT																
	RCT-O																
ROM + Covid vaccine	SR			1						1							
	RCT																
	RCT-O																

Note: RCT = randomized controlled trial; RCT-O = ongoing randomized controlled trial; SR = systematic review; MDT = rifampicin, dapsone and clofazimine; ROM = rifampicin, ofloxacin and minocycline.

[1] Bacillary index = Measurement of the density of bacilli and the proportion of viable M. leprae in samples;

[2] Clinical improvement = Disappearance or size reduction of the lesion, along with decreased hypopigmentation, erythema, infiltration, and improvement or cessation of local symptoms;

[3] Cure = Complete resolution of the lesion without evidence of clinical persistence;

[4] Proinflammatory cytokines = Assessment of cellular proliferation and IFN-γ production levels;

[5] Serological response = Measurement and quantification of antibody titles;

[6] Erythema nodosum leprosum = Assessment of the frequency and severity of ENL episodes, including changes in their duration;

[7] Liver function = Measurement of serum bilirubin, serum glutamic pyruvic transaminase (ALT), and serum glutamic oxalacetic transaminase (AST);
[8] Neuritis = Presence of pain during the clinical interview or patient-reported pain in one or more peripheral nerve trunks during the treatment period;
[9] Reversal reactions = Occurrence and classification of reversal reactions as Type 1 or Type 2.
The number inside the boxes indicate the number of studies assessing each drug/outcome.
Quality assessment of systematic reviews: ■ Critically low; ■ Low; ■ Moderate; ■ High quality.
Risk of bias assessment of RCTs: ■ High; ■ Moderate; ■ Low risk of bias.

Supplementary Figure 13 - EGM of Lymphatic filariasis

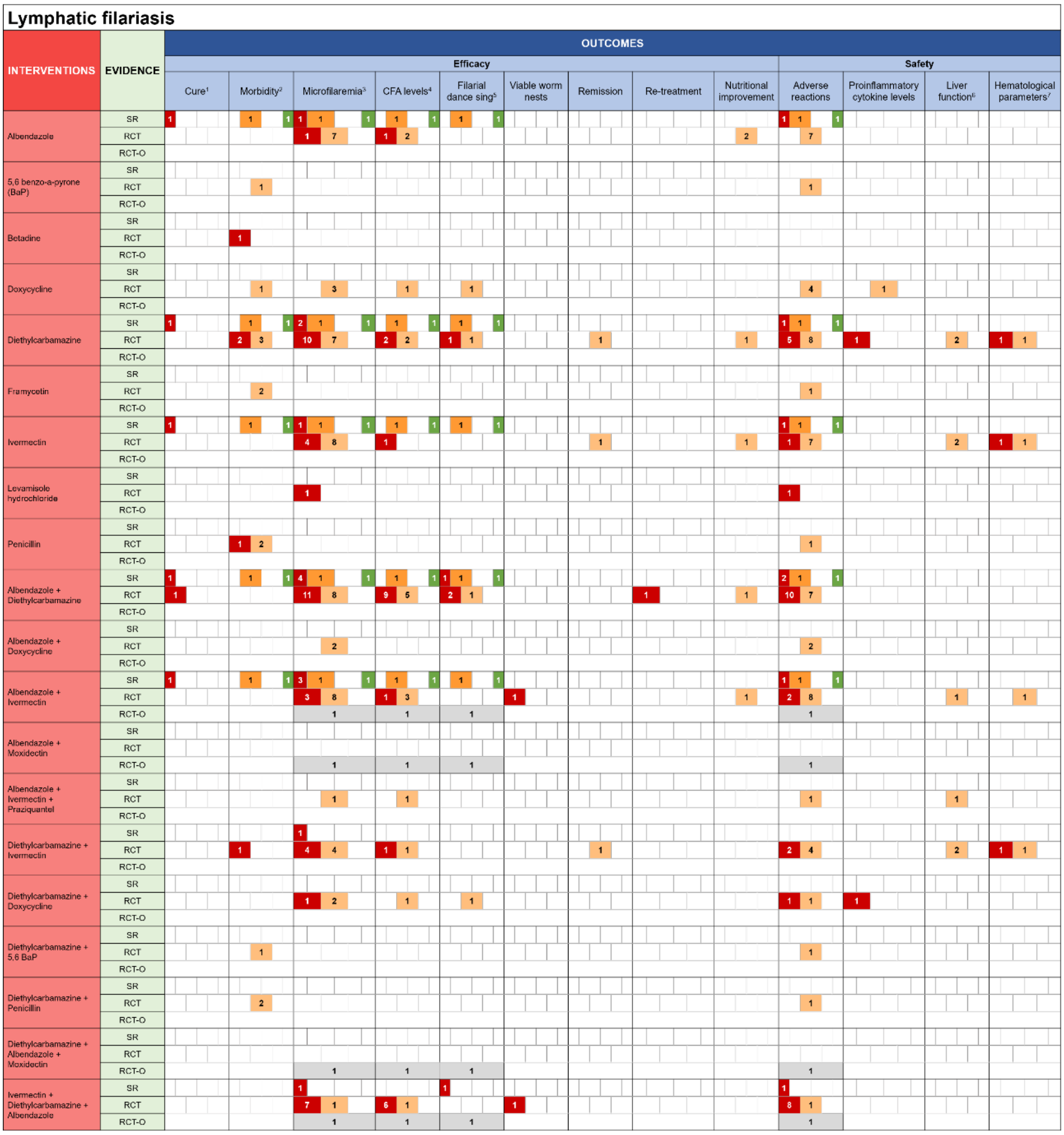

Note: RCT = randomized controlled trial; RCT-O = ongoing randomized controlled trial; SR = systematic review.
¹ Cure = Complete clearance of microfilaremia and filarial antigenemia assessed 12 months post-treatment;
² Morbidity = Occurrence of lymphoedema and frequency of adenolymphangitis attacks;
³ Microfilaremia = Assessment of microfilariae levels, total counts, and the overall rate of microfilaremia;
⁴ CFA levels = Measurement of antigenemia and circulating filarial antigen (CFA) concentration;
⁵ Filarial dance sign = Detection of adult worm presence and movement;
⁶ Liver function = Measurement of serum creatinine, total bilirubin, aspartate aminotransferase (SGOT), alanine aminotransferase (SGPT), and alkaline phosphatase (AP) levels;
⁷ Hematological parameters = Assessment of blood profile through a complete blood cell count.
The number inside the boxes indicate the number of studies assessing each drug/outcome.
Quality assessment of systematic reviews: ■ Critically low; ■ Low; ■ Moderate; ■ High quality.
Risk of bias assessment of RCTs: ■ High; ■ Moderate; ■ Low risk of bias.

Supplementary Figure 14 - EGM of Mycetoma, chromoblastomycosis and other deep mycoses

Mycetoma

INTERVENTIONS	EVIDENCE	OUTCOMES				
		Efficacy				
		Disappearance of itching	Negativity of culture	Healing of lesion[1]	Recurrence	Mortality
5-flurouracil	SR					
	RCT	1	1	1	1	
	RCT-O					
Ajoene	SR					
	RCT	1	1	1	1	
	RCT-O					
Amphotericin B	SR			1	1	1
	RCT					
	RCT-O					
Dapsone	SR			1	1	1
	RCT					
	RCT-O					
Doxycycline with wound excision	SR			1	1	1
	RCT					
	RCT-O					
Fluconazole with wound excision	SR			1	1	1
	RCT					
	RCT-O					
Itraconazole with and without wound excision	SR			1	1	1
	RCT					
	RCT-O					
Ketoconazole	SR			1	1	1
	RCT					
	RCT-O					
Miconazole with wound excision	SR			1	1	1
	RCT					
	RCT-O					
Tetracycline with or without amputation	SR			1	1	1
	RCT					
	RCT-O					
Trimethoprim	SR			1	1	1
	RCT					
	RCT-O					
Voriconazole	SR			1	1	1
	RCT					
	RCT-O					

Mycetoma (continued)

INTERVENTIONS	EVIDENCE	OUTCOMES: Efficacy – Disappearance of itching	Negativity of culture	Healing of lesion[1]	Recurrence	Mortality
Amikacin + Dapsone	SR			1	1	1
	RCT					
	RCT-O					
Amikacin + Sulphametoxazole-Trimethoprim	SR			1	1	1
	RCT					
	RCT-O					
Amoxilin + Itraconazole + Fluconazole + Terbinafine followed by surgery	SR			1	1	1
	RCT					
	RCT-O					
Amphotericin B + Itraconazole	SR			1	1	1
	RCT					
	RCT-O					
Amphotericin B + Ketoconazole + Flucytosine + Fluconazole	SR			1	1	1
	RCT					
	RCT-O					
Cotrimoxazole + Ketoconazole	SR			1	1	1
	RCT					
	RCT-O					
Cotrimoxazole + Voriconazole + Caspogugin	SR			1	1	1
	RCT					
	RCT-O					
Dapsone + Sulphametoxazole-Trimethoprim + Sulfadiazine	SR			1	1	1
	RCT					
	RCT-O					
Itraconazole + Flucytosine	SR			1	1	1
	RCT					
	RCT-O					
Minocycline + Sulphametoxazole-Trimethoprim with surgery	SR			1	1	1
	RCT					
	RCT-O					
Sulphametoxazole-Trimethoprim	SR			1	1	1
	RCT					
	RCT-O					
Sulphametoxazole-Trimethoprim + Dapsone + Rifampicin	SR			1	1	1
	RCT					
	RCT-O					
Sulphametoxazole-Trimethoprim + Itraconazole	SR			1	1	1
	RCT					
	RCT-O					
Sulphametoxazole-Trimethoprim + Rifampicin with wound excision	SR			1	1	1
	RCT					
	RCT-O					
Voriconazole + Itraconazole + Cotrimoxazole	SR			1	1	1
	RCT					
	RCT-O					

Note: RCT = randomized controlled trial; RCT-O = ongoing randomized controlled trial; SR = systematic review.
[1] Healing of lesion = Amelioration of keratotic crusting or scarring of the existing lesions.
The number inside the boxes indicate the number of studies assessing each drug/outcome.
Quality assessment of systematic reviews: ■ Critically low; ■ Low; ■ Moderate; ■ High quality.
Risk of bias assessment of RCTs: ■ High; ■ Moderate; ■ Low risk of bias.

Supplementary Figure 15 - EGM of Onchocerciasis (river blindness)

Onchocerciasis

INTERVENTIONS	EVIDENCE	OUTCOMES								
		Efficacy					Safety			
		Microfilaremia[1]	Viability and fertility of adult worms[2]	Number of nodules	Visual acuity[3]	Reconstruction of skin microfilaria	Adverse reactions	Mazzott-type reaction	Ocular adverse events	Proinflammatory cytokines levels
Albendazole	SR									
	RCT	1, 1	1, 1	1			1, 1			
	RCT-O									
Amocarzine	SR									
	RCT	1	1	1			1	1	1	
	RCT-O									
Doxycycline	SR									
	RCT	1, 2	2, 1	1		1, 1	2, 2			
	RCT-O									
Diethylcarbamazine	SR									
	RCT	2					1		2	
	RCT-O									
Emodepside	SR									
	RCT									
	RCT-O	1	1			1				
Ivermectin	SR	1			1		1			
	RCT	2, 7	2, 3	4	1, 1	1	2, 6	1	2, 3	1
	RCT-O	3	2			1	2			
Levamisole	SR									
	RCT	1	1	1			1			
	RCT-O									
Metrifonate	SR									
	RCT	1				1	1			
	RCT-O									
Minocycline	SR									
	RCT	1	1				1			
	RCT-O									
Moxidectin	SR									
	RCT									
	RCT-O	2	1				2			
Amocarzine + Ivermectin	SR									
	RCT	1	1	1			1	1	1	
	RCT-O									
Azithromycin + Ivermectin	SR									
	RCT	1	1							
	RCT-O									
Azithromycin + Rifampicin + Ivermectin	SR									
	RCT	1	1							
	RCT-O									
Doxycycline + Albendazole	SR									
	RCT	1	1				1			
	RCT-O									
Doxycycline + Ivermectin	SR	1			1		1			
	RCT		1			1	1			
	RCT-O									
Ivermectin + Albendazole	SR									
	RCT	2	2	1			2			
	RCT-O	1	1				1		1	
Ivermectin + Diethylcarbamazine + Albendazole	SR									
	RCT	1	1				1			
	RCT-O	1	1				1		1	
Levamisole + Albendazole	SR									
	RCT	1	1	1			1			
	RCT-O									
Levamisole + Ivermectin	SR									
	RCT	1	1	1			1			
	RCT-O									
Moxidectin + Albendazole	SR									
	RCT									
	RCT-O	1	1				1		1	
Moxidectin + Diethylcarbamazine + Albendazole	SR									
	RCT									
	RCT-O	1	1				1		1	
Rifampicin + Ivermectin	SR									
	RCT	1	1							
	RCT-O									

Note: RCT = randomized controlled trial; RCT-O = ongoing randomized controlled trial; SR = systematic review.

[1] Microfilaremia = Measurement of microfilaria load and circulating levels of microfilaremia;

[2] Viability and fertility of adult worms = Determination of adult worm mortality and reproductive status;

[3] Visual acuity = Clinical evaluation of the optic disc, chroidoretinal health, and retinal vasculature.

The number inside the boxes indicate the number of studies assessing each drug/outcome.

Quality assessment of systematic reviews: ■ Critically low; ■ Low; ■ Moderate; ■ High quality.

Risk of bias assessment of RCTs: ■ High; ■ Moderate; ■ Low risk of bias.

Supplementary Figure 16 - EGM of Rabies

Rabies

INTERVENTIONS	EVIDENCE	OUTCOMES: Efficacy – Mortality	Efficacy – Immunogenicity[1]	Efficacy – Virus neutralizing antibody[2]	Safety – Adverse reactions	Safety – Serious Adverse Events
Chromatographically purified rabies vaccine (CPRV)	SR					
	RCT				1	
	RCT-O					
Equine rabies immunoglobulin injection	SR					
	RCT					
	RCT-O			1		
Freeze-dried human rabies vaccine (Vero cells)	SR					
	RCT					
	RCT-O		1		1	1
Human diploid cell culture rabies vaccine (HDCV, Imovax)	SR					
	RCT		1		1	
	RCT-O					
Human diploid cell strain vaccine (HDCSV)	SR					
	RCT		1		2	
	RCT-O					
Rabies Human Immunoglobulin (HRIG)	SR					
	RCT					
	RCT-O		1		1	
Natural full human monoclonal antibody CBB 1 injection	SR					
	RCT					
	RCT-O		1		1	
Purified chick embryo cell rabies vaccine (VaxiRab N)	SR					
	RCT		1		1	
	RCT-O					
Purified chick embryo cell vaccine (PCEC, Rabipur)	SR		1		1	
	RCT		5 1	1	6 1	
	RCT-O		1		1	1
Purified vero cell rabies vaccine (Rabivax-S)	SR					
	RCT		1		1	
	RCT-O		1			
Rabies vaccine Rabix-VC	SR					
	RCT					
	RCT-O		1		1	1
Recombinant human monoclonal antibody (SII RMAb)	SR					
	RCT			1	1	
	RCT-O					
Vero cell rabies vaccine (PVRV, Verorab)	SR					
	RCT		1 1	1	3 1	
	RCT-O			1	1	1
ChAdOx2 RabG + Inactivated Rabies Vaccine	SR					
	RCT					
	RCT-O			1	1	1
Chromatographically purified rabies vaccine (CPRV) + Human rabies immunoglobulin (HRIG)	SR					
	RCT		1	1	1	
	RCT-O					
Human diploid cell strain vaccine (HDCSV) + Aluminium hydroxide	SR					
	RCT		1		1	
	RCT-O					
Human diploid cell strain vaccine (HDCSV) + Human rabies immune globulin (HRIG)	SR					
	RCT		2		2	
	RCT-O					
Human diploid cell vaccine (HDCV) + Human rabies immunoglobulin (HRIG)	SR					
	RCT		1	1 1	1 1	
	RCT-O					

Note: RCT = randomized controlled trial; RCT-O = ongoing randomized controlled trial; SR = systematic review.

[1] Immunogenicity = Measurement of rabies virus neutralizing antibody (RVNA) response, expressed as geometric mean titers and individual RVNA titers;

[2] Virus neutralizing antibody = Determination of the geometric mean titers (GMT) of rabies virus neutralizing antibodies.

The number inside the boxes indicate the number of studies assessing each drug/outcome.

Quality assessment of systematic reviews: ■ Critically low; ■ Low; ■ Moderate; ■ High quality.

Risk of bias assessment of RCTs: ■ High; ■ Moderate; ■ Low risk of bias.

Supplementary Figure 17 - EGM of Scabies and others ectoparasitoses

Scabies and other ectoparasitoses

INTERVENTIONS	EVIDENCE	OUTCOMES: Efficacy – Cure[1]	Morbidity[2]	Pruritus[3]	Improvement of the infection[4]	Density of ectoparasite	Treatment failure[5]	Severity of skin lesion	Re-treatment	Re-infestition	Safety – Adverse reactions	Treatment discontinuation	Macrolide resistance
Benzyl-benzoate	SR	1	1	1						1	1, 1		
	RCT	1, 2		1	1	1					1, 1		
	RCT-O												
Crotamiton	SR	1		1						1	1		
	RCT	4, 1					3, 1				3		
	RCT-O												
Gamma benzene hexachloride	SR												
	RCT	1		1							1		
	RCT-O												
Ivermectin	SR	2	1, 1	1					1	1	1, 2	1	
	RCT	10, 2	3	2, 3	1, 2	1	8	1		1	9, 2		1
	RCT-O			1	3						2		
Lindane	SR	1		1						1	1		
	RCT	2			1, 1		2				1		
	RCT-O												
Malathion	SR	1		1						1	1		
	RCT												
	RCT-O												
Moxidectin	SR												
	RCT												
	RCT-O	1									1		
Permethrin	SR	2	1, 1	1					1	1	1, 2	1	
	RCT	8, 4	2	3, 2	2	1	5, 1	1			3, 2		
	RCT-O			1							1		
Spinosad	SR												
	RCT	1			1	1					1		
	RCT-O												
Sulfur	SR	1		1						1	1		
	RCT	1					1			1	1		
	RCT-O												
Synergized pyrethrins	SR	1		1						1	1		
	RCT	1		1	1								
	RCT-O												
Diethylcarbamazine + Albendazole	SR												
	RCT										1		
	RCT-O												
Ivermectin + Albendazole	SR		1, 1								1		
	RCT												
	RCT-O												
Ivermectin + Azithromycin	SR												
	RCT		1										1
	RCT-O												
Ivermectin + Diethylcarbamazine + Albendazole	SR												
	RCT										1		
	RCT-O												
Ivermectin + Permethrin	SR	1		1						1	1		
	RCT	1		1							1		
	RCT-O												

Note: RCT = randomized controlled trial; RCT-O = ongoing randomized controlled trial; SR = systematic review.

[1] Cure = Achieving clinical and microscopic cure, characterized by the healing of all existing lesions and the absence of new lesions

[2] Morbidity = Occurrence or clinical presentation of impetigo and eczema;

[3] Pruritus = Assessment of clinical improvement or reduction in itching intensity

[4] Improvement of the infection = Evaluation of the persistence of signs and symptoms during or after treatment, including the assessment of incomplete healing of lesions;

[5] Treatment failure = Detection or clinical presence of new lesions following the completion of treatment.

The number inside the boxes indicate the number of studies assessing each drug/outcome.

Quality assessment of systematic reviews: ■ Critically low; ■ Low; ■ Moderate; ■ High quality.

Risk of bias assessment of RCTs: ■ High; ■ Moderate; ■ Low risk of bias.

Supplementary Figure 18 - EGM of Schistosomiasis

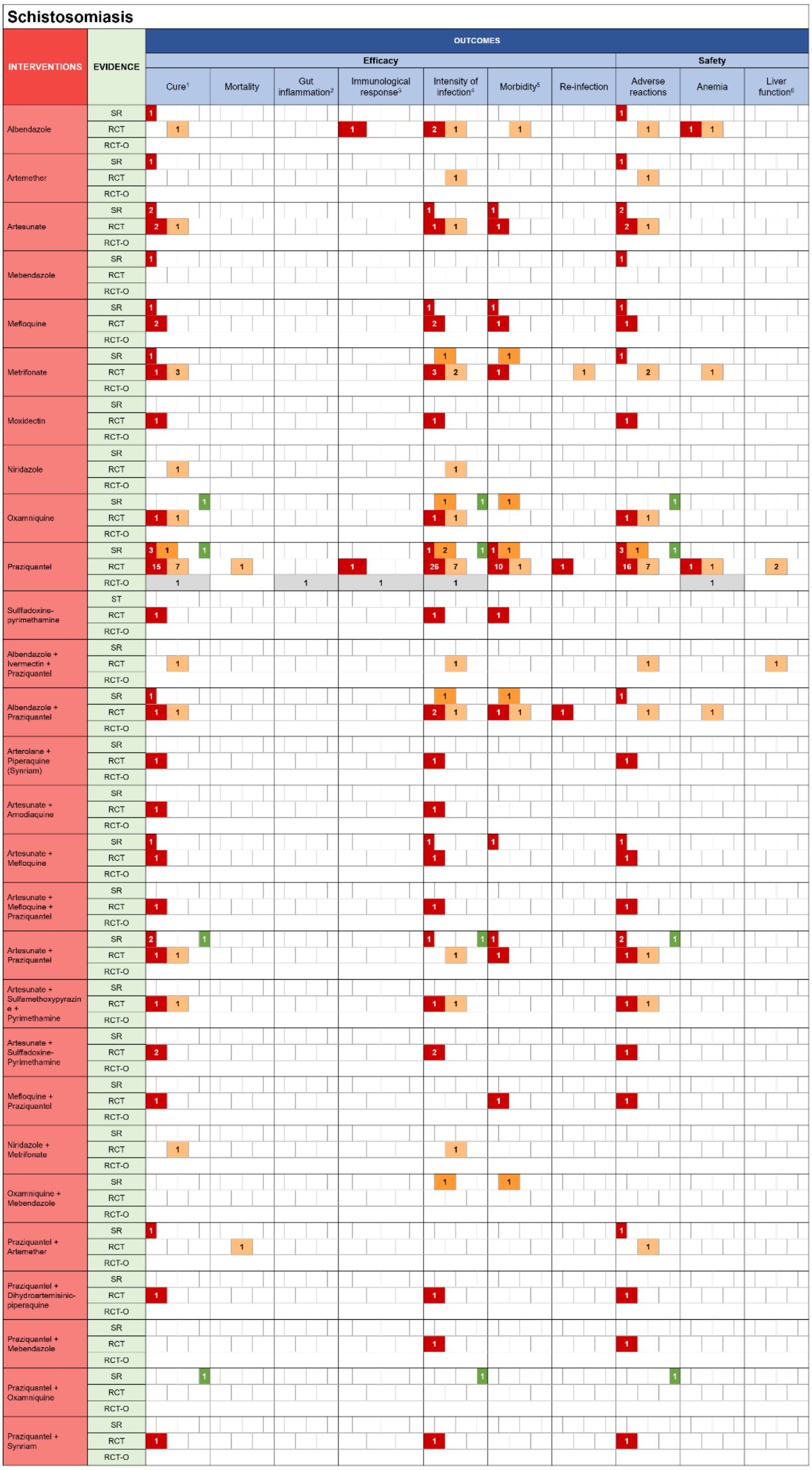

Note: RCT = randomized controlled trial; RCT-O = ongoing randomized controlled trial; SR = systematic review.

[1] Cure = Evaluation of the cure rate based on egg reduction, confirmation of three consecutive urine samples negative for eggs, and the cessation of viable egg excretion in stools;

[2] Gut inflammation = Assessment of intestinal inflammation via measurement of fecal calprotectin levels;

³ Immunological response = Evaluation of cytokine and antibody responses to schistosome worm antigen (SWA) and soluble egg antigen (SEA), including measurement of C-reactive protein (CRP), serum IL-6, and serum TNF-alpha levels;
⁴ Intensity of infection = Quantitative assessment based on parasite egg counts per gram of sample;
⁵ Morbidity = Clinical presentation of urinary tract infection, hematuria, proteinuria, hepatomegaly, or splenomegaly;
⁶ Liver function = Measurement of serum bilirubin, serum glutamic pyruvic transaminase (SGPT/ALT), and serum glutamic oxalacetic transaminase (SGOT/AST) levels.
The number inside the boxes indicate the number of studies assessing each drug/outcome.
Quality assessment of systematic reviews: ■ Critically low; ■ Low; ■ Moderate; ■ High quality.
Risk of bias assessment of RCTs: ■ High; ■ Moderate; ■ Low risk of bias.

Supplementary Figure 19 - EGM of Snakebite envenoming

Snakebite

INTERVENTIONS		EVIDENCE	OUTCOMES				
			Efficacy		Safety		
			Mortality	Snakebite severity score	Adverse reactions	Limb function[1]	Clotting functions[2]
Treatment Against snakebite	Anti-snake venenum	SR					
		RCT	1		2	2	2
		RCT-O					
	Crotalidae polyvalent immune Fab (ovine)	SR			1		
		RCT			1	1	
		RCT-O					
	Lyophilized monovalent antivenom alone or with Heparin	SR					
		RCT					1
		RCT-O					
	Varespladib	SR					
		RCT					
		RCT-O	1	1	1	1	1

Note: RCT = randomized controlled trial; RCT-O = ongoing randomized controlled trial; SR = systematic review.
¹ Limb function = Assessment of systemic impact through the Snakebite Severity Score, evaluating limb-related functions across the pulmonary, cardiovascular, hematologic, renal, and nervous systems;
² Clotting functions = Evaluation of clotting time and identification of clinical abnormalities in the coagulation profile.
The number inside the boxes indicate the number of studies assessing each drug/outcome.
Quality assessment of systematic reviews: ■ Critically low; ■ Low; ■ Moderate; ■ High quality.
Risk of bias assessment of RCTs: ■ High; ■ Moderate; ■ Low risk of bias.

Supplementary Figure 20 - EGM of Snakebite envenoming (anti-venom serum)

Snakebite

INTERVENTIONS		EVIDENCE	OUTCOMES				
			Efficacy		Safety		
			Mortality	Snakebite severity score	Adverse reactions	Limb function	Clotting functions
Treatment of adverse events to antivenenum	Adrenaline	SR			1		
		RCT			2		
		RCT-O					
	Hydrocortisone	SR			1		
		RCT			1 2		
		RCT-O					
	Promethazine	SR					
		RCT			1 1		
		RCT-O					
	Chloramphenicol	SR					
		RCT			1		
		RCT-O					
	Adrenaline + Hydrocortisone	SR					
		RCT			1		
		RCT-O					
	Adrenaline + Promethazine	SR					
		RCT			1		
		RCT-O					
	Adrenaline + Hydrocortisone + Promethazine	SR					
		RCT			1		
		RCT-O					
	Hydrocortisone + Chlorpheniramine	SR			1		
		RCT			1		
		RCT-O					
	Hydrocortisone + Diphenhydramine	SR			1		
		RCT					
		RCT-O					
	Gentamicin + Chloranphenicol	SR					
		RCT			1		
		RCT-O					

Note: RCT = randomized controlled trial; RCT-O = ongoing randomized controlled trial; SR = systematic review.
[1] Limb function = Assessment of systemic impact through the Snakebite Severity Score, evaluating limb-related functions across the pulmonary, cardiovascular, hematologic, renal, and nervous systems;
[2] Clotting functions = Evaluation of clotting time and identification of clinical abnormalities in the coagulation profile.
The number inside the boxes indicate the number of studies assessing each drug/outcome.
Quality assessment of systematic reviews: ■ Critically low; ■ Low; ■ Moderate; ■ High quality.
Risk of bias assessment of RCTs: ■ High; ■ Moderate; ■ Low risk of bias.

Supplementary Figure 21 - EGM of Soil-transmitted helminthiases

Soil-transmitted helminthiases

INTERVENTIONS	EVIDENCE	OUTCOMES						
		Efficacy					Safety	
		Antopometric improvement[1]	Cure[2]	Intensity of infection[3]	Nutritional improvement	Re-infection	Adverse reactions	Liver function[4]
Albendazole	SR		3 1	2 1			1 1	
	RCT	1 1	9 10 4	10 10 4	2	2 1	3 10 3	1
	RCT-O		2	2			2	
Bephenium-hydroxynapthoate	SR		1				1	
	RCT							
	RCT-O							
Diethylcarbamazine	SR		1				1	
	RCT	1	1	1 1	1		1	
	RCT-O							
Emodepside	SR							
	RCT		1	1			1	
	RCT-O							
Flubendazole	SR		1				1	
	RCT							
	RCT-O							
l-Tetramisole	SR		1				1	
	RCT							
	RCT-O							
Ivermectin	SR		2 1	1 1			1 1	
	RCT		2 1	2 2	1	1	1 1	
	RCT-O							
Levamisole	SR		2	1			1	
	RCT		1	1				
	RCT-O							
Mebendazole	SR		3 1	2 1			1 1	
	RCT		5 4 2	5 4 2		1	1 2 2	
	RCT-O							
Metronidazole	SR							
	RCT		1				1	
	RCT-O							
Moxidectin	SR		1				1	
	RCT		2	2			2	
	RCT-O							
Nitazoxanide	SR		1 1	1 1			1	
	RCT		1	1			1	
	RCT-O							
Oxfendazole	SR							
	RCT							
	RCT-O		2	2			2	
Oxantel pamoate	SR		1	1				
	RCT		1	1			1	
	RCT-O							
Oxantel-pyrantel	SR		2	1			1	
	RCT							
	RCT-O							
Piperazine citrate	SR							
	RCT	1	1	1		1		
	RCT-O							
Praziquantel	SR		1				1	
	RCT			1			1	
	RCT-O							
Pyrantel pamoate	SR		2	1			1	
	RCT	1		1				
	RCT-O							
Thiabendazole	SR		1				1	
	RCT							
	RCT-O							
Tribendimidine	SR		1	1				
	RCT		1	1 1			1 2	
	RCT-O							
ZP5-9676	SR							
	RCT							
	RCT-O		1	1				

Soil-transmitted helminthiases (continued)

INTERVENTIONS	EVIDENCE	OUTCOMES																				
		Efficacy															Safety					
		Antopometric improvement[1]			Cure[2]			Intensity of infection[3]			Nutritional improvement			Re-infection			Adverse reactions			Liver function[4]		
Albendazole + Diethylcarbamazine	SR				3			2									1					
	RCT		1		2			2	1			1						1				
	RCT-O																					
Albendazole + Ivermectin	SR				3	1		2	1								1	1				
	RCT				3	1	4	3	2	4		1			2	1	1		3			
	RCT-O																					
Albendazole + Ivermectin + Praziquantel	SR																					
	RCT					1												1			1	
	RCT-O																					
Albendazole + Levamisole	SR				1			1														
	RCT																					
	RCT-O																					
Albendazole + Mebendazole	SR				2			2														
	RCT				1	1	2	1	1	2					1				2			
	RCT-O																					
Albendazole + Nitazoxanide	SR				2			2														
	RCT					1			1									1				
	RCT-O																					
Albendazole + Oxantel pamoate	SR				2			2														
	RCT				1	2	1	1	2	1					1		1	1	1			
	RCT-O																					
Albendazole + Praziquantel	SR				2			1									1					
	RCT				1			1									1					
	RCT-O																					
Albendazole + Pyrantel pamoate	SR																					
	RCT						1			1									1			
	RCT-O																					
Albendazole + Tribendimidine	SR																					
	RCT				1			1									1					
	RCT-O																					
Levamisole + Mebendazole	SR				2			1									1					
	RCT					1			1													
	RCT-O																					
Mebendazole + Ivermectin	SR				2			2														
	RCT						1			1									1			
	RCT-O																					
Mebendazole + Pyrantel pamoate	SR				1			1														
	RCT																					
	RCT-O																					
Moxidectin + Albendazole	SR				2			1									1					
	RCT				2			2									2					
	RCT-O																					
Moxidectin + Tribendimidine	SR				2			1									1					
	RCT				1			1									1					
	RCT-O																					
Praziquantel + Mebendazole	SR																					
	RCT							1									1					
	RCT-O																					
Tribendimidine + Ivermectin	SR				1			1														
	RCT				1			1									1					
	RCT-O																					
Tribendimidine + Oxantel pamoate	SR				1			1														
	RCT				1			1									1					
	RCT-O																					
Albendazole +Pyrantel-Oxantel pamoate	SR				1			1														
	RCT																					
	RCT-O																					

Note: RCT = randomized controlled trial; RCT-O = ongoing randomized controlled trial; SR = systematic review.

[1] Anthropometric improvement = Evaluation of physical growth and body composition through measurements of weight, height, triceps skinfold, mid-arm circumference, and chest circumference;

[2] Cure = Achievement of parasitological cure, defined by negative egg counts following the completion of treatment;

[3] Intensity of infection = Quantitative assessment based on parasite egg counts and the calculated percentage of egg reduction;

[4] Liver function = Measurement of serum bilirubin, serum glutamic pyruvic transaminase (SGPT/ALT), and serum glutamic oxalacetic transaminase (SGOT/AST) levels.
The number inside the boxes indicate the number of studies assessing each drug/outcome.
Quality assessment of systematic reviews: ■ Critically low; ■ Low; ■ Moderate; ■ High quality.
Risk of bias assessment of RCTs: ■ High; ■ Moderate; ■ Low risk of bias.

Supplementary Figure 22 - EGM of Taeniasis/Cysticercosis

Taeniasis/Cysticercosis

INTERVENTIONS	EVIDENCE	OUTCOMES										
		Efficacy									Safety	
		Cyst control[1]	Hospitalization	Lesion calcification	Lesion resolution[2]	Morbidity[3]	Mortality	Resolution of symptoms	Seizure control	Seizure recurrence	Adverse reactions	Treatment discontinuation
Albendazole	SR	1 1	1	1 1	2 1 2	1 1	1 1	1	1 1	2 1 2	2 2	
	RCT	2 2		2 1	5 2				1 4	4	3 3	
	RCT-O											
Carbamazepine	SR		1				1			1	1	1
	RCT			1	2				1	1	1	
	RCT-O											
Levetiracetam	SR											
	RCT				1				1		1	
	RCT-O											
Methylprednisone	SR			1	1					1		
	RCT											
	RCT-O											
Oxfendazole	SR											
	RCT											
	RCT-O	1		1	1				1	1	1	
Phenytoin	SR		1				1			1	1	1
	RCT			1	1					1		
	RCT-O											
Praziquantel	SR	1 1	1	1	2 1	1 1	1 1	1	1 1	2 1	2 1	
	RCT	2			2				1		1	
	RCT-O											
Prednisone	SR											
	RCT	1							1	1	1	
	RCT-O											
Prednisolone	SR	1	1	1 1	2 1 2	1	1	1	1 1	1 1 2	1 2	
	RCT	1		1 2	1 1				1 1	1 2		
	RCT-O											
Albendazole + Antiepileptic*	SR											
	RCT					1			1	1	1	
	RCT-O											
Albendazole + Dexamethasone	SR	1	1	1	1 1 2	1	1	1	1	1 1 2	1 2	
	RCT	1			1				1	1	2 1	
	RCT-O											
Albendazole + Praziquantel	SR											
	RCT	1 1			1				1 1	1	1 1	
	RCT-O	1							1			
Albendazole + Prednisolone	SR	1	1	1 1	2 1 2	1	1	1	1 1	1 1 2	1 2	
	RCT			1	1					1		
	RCT-O											
Albendazole + Prednisone	SR											
	RCT	1 1							1 1	1	1	
	RCT-O											
Carbamazepine + Phenytoin	SR		1				1			1	1	1
	RCT											
	RCT-O											
Praziquantel + Cimetidine	SR											
	RCT	1			1							
	RCT-O											
Prednisolone + Antiepileptic*	SR											
	RCT				1					1 1		
	RCT-O											
Prednisolone + Praziquantel	SR				1					1	1	
	RCT											
	RCT-O											

Note: RCT = randomized controlled trial; RCT-O = ongoing randomized controlled trial; SR = systematic review; Antiepileptic = phenytoin or carbamazepine.

[1] Cyst control = Clinical monitoring of the disappearance of cysts or a significant reduction in the total number of cysts;

[2] Lesion reduction = Radiographic assessment of lesion disappearance as confirmed by computed tomography (CT) or magnetic resonance imaging (MRI);

[3] Morbidity = Clinical presentation of neurological complications, including the presence of gliosis, edema, raised intracranial pressure or intracranial hypertension, emergence of fresh symptoms, and resulting disability.

The number inside the boxes indicate the number of studies assessing each drug/outcome.

Quality assessment of systematic reviews: ■ Critically low; ■ Low; ■ Moderate; ■ High quality.

Risk of bias assessment of RCTs: ■ High; ■ Moderate; ■ Low risk of bias.

Supplementary Figure 23 - EGM of Trachoma

Trachoma

INTERVENTIONS	EVIDENCE	OUTCOMES								
		Efficacy						Safety		
		Active trachoma	Cure¹	Mortality	Recurrence	Re-emergence	Treatment failure²	Adverse reactions	Antibiotic resistance	Severity of recurrence
Azithromycin	SR	2	2				1	2, 1	2, 1	
	RCT		2, 3, 1	1	1, 2	1, 1, 1		4, 2		
	RCT-O									
Cefotetan	SR		1							
	RCT									
	RCT-O									
Cefoxitin	SR		1							
	RCT									
	RCT-O									
Ceftizoxime	SR		1							
	RCT									
	RCT-O									
Chloramphenicol	SR									
	RCT		1				1			
	RCT-O									
Ciprofloxacin	SR		1							
	RCT									
	RCT-O									
Clindamycin	SR									
	RCT		1					1		
	RCT-O									
Doxycycline	SR	2	1				1	1, 1	1	
	RCT									
	RCT-O									
Erythromycin	SR	1	2				1	2	2	
	RCT		2				1	1		
	RCT-O									
Levofloxacin	SR		1				1	1		
	RCT									
	RCT-O									
Minocycline	SR									
	RCT		1							
	RCT-O									
Ofloxacin	SR		1							
	RCT									
	RCT-O									
Oxytetracycline	SR	2						1	1	
	RCT									
	RCT-O									
Piperacillin	SR		1							
	RCT									
	RCT-O									
Pivmecillinam hydrochloride	SR		1				1	1		
	RCT									
	RCT-O									
Povidone-iodine	SR									
	RCT		1							
	RCT-O									
Rifalazil	SR		1				1	1		
	RCT									
	RCT-O									
Sitafloxacin	SR		1				1	1		
	RCT									
	RCT-O									
Sulphadimethoxine	SR	2						1	1	
	RCT									
	RCT-O									

Trachoma (continued)

INTERVENTIONS	EVIDENCE	OUTCOMES								
		Efficacy						Safety		
		Active trachoma	Cure[1]	Mortality	Recurrence	Re-emergence	Treatment failure[2]	Adverse reactions	Antibiotic resistance	Severity of recurrence
Sulphafurazole	SR	2						1	1	
	RCT									
	RCT-O									
Sulphamethoxypyridazine	SR	2						1	1	
	RCT									
	RCT-O									
Tetracycline	SR	2						1	1	
	RCT		2, 1, 1		1, 2	1		1		1, 2
	RCT-O									
Trisulphapyrimidines	SR	2						1	1	
	RCT									
	RCT-O									
Amoxicillin + Clavulanic acid	SR		1							
	RCT									
	RCT-O									
Amoxicillin + Clavulanic acid + Doxycyline	SR		1							
	RCT									
	RCT-O									
Aztreonam + Clindamycin	SR		1							
	RCT									
	RCT-O									
Cefotaxime + Tetracycline	SR		1							
	RCT									
	RCT-O									
Cefotetan + Doxycycline	SR		1							
	RCT									
	RCT-O									
Cefoxitin + Doxycycline	SR		1							
	RCT									
	RCT-O									
Cefoxitin + Probenecid + Doxycycline	SR		1							
	RCT									
	RCT-O									
Ceftizoxime + Doxycycline	SR		1							
	RCT									
	RCT-O									
Ceftizoxime + Tetracycline	SR		1							
	RCT									
	RCT-O									
Clindamycin + Amikacin	SR		1							
	RCT									
	RCT-O									
Clindamycin + Gentamicin	SR		1							
	RCT									
	RCT-O									
Clindamycin + Tobramycin	SR		1							
	RCT									
	RCT-O									
Erythromycin + Tetracycline	SR	1								
	RCT									
	RCT-O									
Metronidazole + Doxycycline	SR		1							
	RCT									
	RCT-O									
Metronidazole + Gentamicin	SR		1							
	RCT									
	RCT-O									
Metronidazole + Gentamicin + Doxycycline	SR		1							
	RCT									
	RCT-O									

Trachoma (continued)

INTERVENTIONS	EVIDENCE	OUTCOMES								
		Efficacy						Safety		
		Active trachoma	Cure¹	Mortality	Recurrence	Re-emergence	Treatment failure²	Adverse reactions	Antibiotic resistance	Severity of recurrence
Metronidazole + Penicillin	SR		1							
	RCT									
	RCT-O									
Oxytetracycline + Polymyxin	SR	1						1	1	
	RCT		1 1			1		1		
	RCT-O									
Penicillin + Doxycycline	SR		1							
	RCT									
	RCT-O									
Sulbactam + Ampicillin	SR		1							
	RCT									
	RCT-O									
Sulbactam + Ampicillin + Doxycycline	SR		1							
	RCT									
	RCT-O									
Sulbactam + Ampicillin + Tetracycline	SR		1							
	RCT									
	RCT-O									
Sulphametoxazole + Trimethoprim	SR		1							
	RCT									
	RCT-O									
Sulphafurazole + Sulphadimethoxine	SR	2						1	1	
	RCT									
	RCT-O									
Tetracycline + Polymyxin	SR	1								
	RCT									
	RCT-O									
Ticarcillin + Clavulanic acid	SR		1							
	RCT									
	RCT-O									

Note: RCT = randomized controlled trial; RCT-O = ongoing randomized controlled trial; SR = systematic review.
¹ Cure = Clinical and/or parasitological cure, characterized by the complete resolution of clinical signs;
² Treatment failure = Clinical deterioration characterized by the progression of conjunctival inflammation.
The number inside the boxes indicate the number of studies assessing each drug/outcome.
Quality assessment of systematic reviews: ■ Critically low; ■ Low; ■ Moderate; ■ High quality.
Risk of bias assessment of RCTs: ■ High; ■ Moderate; ■ Low risk of bias.

Supplementary Figure 24 - EGM of Yaws and other endemic treponematoses

Yaws

INTERVENTIONS	EVIDENCE	OUTCOMES					
		Efficacy				Safety	
		Cure[1]	Healing lesions[2]	Recurrence	History of household exposure	Antibiotic resistance	Adverse reactions
Azithromycin oral single dose	SR						
	RCT	2	2		1		2
	RCT-O						
Azithromycin oral three rounds	SR						
	RCT					1	
	RCT-O						
Linezolid oral	SR						
	RCT						
	RCT-O	1	1	1		1	1

Note: RCT = randomized controlled trial; RCT-O = ongoing randomized controlled trial; SR = systematic review.
[1] Cure = Clinical, serological, and/or molecular cure parameters, including the determination of the overall cure rate;
[2] Healing lesions = Clinical assessment of the complete or partial healing of ulcers and other cutaneous lesions.
The number inside the boxes indicate the number of studies assessing each drug/outcome.
Quality assessment of systematic reviews: ■ Critically low; ■ Low; ■ Moderate; ■ High quality.
Risk of bias assessment of RCTs: ■ High; ■ Moderate; ■ Low risk of bias.
